# TGF-β and IL-2 differentially shape T follicular regulatory cell differentiation and stability in vitro

**DOI:** 10.1038/s41423-026-01440-9

**Published:** 2026-06-25

**Authors:** Luisa Bach, Yinshui Chang, Olin Arteaga Transito, Mohammadamin Ghasemi, Lisa Maria Steinheuer, Teresa Steffen, Elena De Domenico, Thomas Ulas, F. Thomas Wunderlich, Marc D. Beyer, Kevin Thurley, Dirk Baumjohann

**Affiliations:** 1 Medical Clinic III for Oncology, Hematology, Immuno-Oncology and Rheumatology, University Hospital Bonn, University of Bonn, Bonn, Germany; 2 Institute of Experimental Oncology, Biomathematics Division, University Hospital Bonn, University of Bonn, Bonn, Germany; 3 Institute of Molecular Psychiatry, University Hospital Bonn, University of Bonn, Bonn, Germany; 4 Platform for Single-Cell Genomics and Epigenomics at the University of Bonn and the German Center for Neurodegenerative Diseases and West German Genome Center, Bonn, Germany; 5 Bioinformatics & Computational Network Research, Life & Medical Sciences Institute (LIMES), University of Bonn, Bonn, Germany; 6 Max Planck Institute for Metabolism Research, Center for Endocrinology, Diabetes and Preventive Medicine (CEDP), Center for Molecular Medicine Cologne (CMMC), Cologne, Germany; 7 Immunogenomics & Neurodegeneration, Deutsches Zentrum für Neurodegenerative Erkrankungen (DZNE), Bonn, Germany; 8 Bonn Center for Mathematical Life Sciences, University of Bonn, Bonn, Germany

**Keywords:** T follicular regulatory cells, CXCR5, FoxP3, IL-2, c-Maf, Lymphocyte differentiation, Follicular T-helper cells, Regulatory T cells

## Abstract

T follicular helper (Tfh) cells and T follicular regulatory (Tfr) cells play critical roles in regulating the activity of the germinal center (GC), which is essential for the generation of high-affinity antibodies. In the GC, Tfh cells help B cells to proliferate and to differentiate into memory B cells and long-lived plasma cells. In contrast, Tfr cells, a specialized subset of regulatory T cells (Tregs), modulate the humoral immune response by suppressing excessive or autoreactive B-cell activity. Here, we established an in vitro differentiation protocol for mouse CD4⁺ T cells that yielded CXCR5⁺FoxP3⁺ Tfr cells that exhibited a Bcl6^hi^PD-1^hi^CD25^lo^GITR^int^ phenotype and were distinct from Treg and Tfh cells. Functionally, in vitro-generated Tfr cells potently suppressed Tfh cell-driven B-cell class switching to IgG1 and downregulated the expression of B-cell costimulatory ligands. While in vitro-generated *Bcl6*-deficient Tfh cells were impaired in providing help to B cells for efficient class switching to IgG1, in vitro-generated *Bcl6*-deficient Tfr cells failed to inhibit Tfh cell-driven B-cell class switching to IgG1. Mechanistically, we showed that Tfr cells emerged from FoxP3^+^ precursors in low-IL-2 environments through a TGF-β- and c-Maf-dependent pathway, allowing for reprogramming and reinforcement of the follicular regulatory cell program in CD4^+^ T cells in vitro.

## Introduction

The germinal center (GC) is a specialized microanatomical structure within secondary lymphoid organs that is critical for the generation of high-affinity, isotype-switched antibodies and the establishment of long-lasting humoral immunity [1]. Within the GC, T follicular helper (Tfh) cells play a pivotal role in supporting B cells by promoting their survival, proliferation, and differentiation into antibody-secreting plasma or memory B cells [2–5]. Due to the random nature of somatic hypermutation of the variable regions of immunoglobulin genes, the GC reaction needs to be tightly regulated to ensure effective immune responses while maintaining self-tolerance and preventing autoimmunity. This task is aided by T follicular regulatory (Tfr) cells, a specialized subset of FoxP3^+^ regulatory T (Treg) cells that express Tfh cell-associated molecules such as the chemokine receptor CXCR5 and the transcription factor Bcl6, PD-1 and ICOS, as well as Treg-associated molecules such as FoxP3, CD25, CTLA, and GITR [6–8]. The dual lineage identity of Tfr cells is accompanied by dual functionality as critical modulators of the GC response: Initially, Tfr cells were characterized by their ability to constrain GC magnitude, limiting the proliferation of Tfh and GC B cells and thereby controlling overall GC size and serum IgG titers [6–8]. However, other studies have demonstrated that the loss of Tfr cells does not alter GC T and B-cell populations but instead impairs antibody affinity maturation, as mice lacking Tfr cells do generate GCs that produce lower-affinity antibodies [9–11], indicating a critical role in selecting high-affinity B-cell clones. Overall, dysregulation of Tfh and Tfr cells leads to a decreased Tfr/Tfh ratio, which is correlated with increased autoantibody production and is linked to the development and activity of autoimmune diseases [12, 13]. Mechanistically, Tfr cells regulate GC B cells directly through CTLA-4-dependent downregulation of CD80 and CD86 [9, 14] or indirectly through suppression of Tfh proliferation and secretion of IL-21 and IL-4, thereby impairing class-switch recombination and antibody production [15, 16]. Moreover, Tfr cells remodel the epigenetic landscape of cocultured Tfh and B cells, which in turn reshapes their glycolytic and oxidative phosphorylation programs [16]. In addition to negatively regulating the GC response, some studies have shown that IL-10 secretion by Tfr cells induces FOXO1 expression in GC B cells, facilitating their polarization toward a proliferative dark-zone phenotype [17, 18].

In contrast to Tfh cells, which develop from conventional naïve CD4 T cells, it is believed that Tfr cells originate from thymic-derived Tregs, thereby maintaining a TCR repertoire that is predominantly self-reactive, which is consistent with their role in immune tolerance [6, 8, 19]. Nevertheless, there is further evidence that inducible Tfr (iTfr) cells may also be generated from FoxP3-negative precursors in certain immunization contexts [20, 21]. As both Tfh cells and Tfr cells share many phenotypic features, including their common localization in close proximity to B cells, it is very likely that Tfr cells share partially overlapping differentiation pathways with Tfh cells. Similar to the multistep differentiation process of Tfh cells, Tfr cells require priming by dendritic cells [15] that provide cognate signaling through the T-cell receptor (TCR) and costimulatory signals through CD28 and ICOS [6, 22, 23]. Furthermore, SAP-dependent stimulation by B cells is required for full Tfr cell differentiation [6, 15], and together, these interactions drive the upregulation of Bcl6 expression and the homing of Tfr cells to the GC via NFAT2-induced CXCR5 expression [24]. While IL-2Rα (CD25)-mediated IL-2 signaling via STAT5 is crucial for Treg homeostasis [25], it acts as a barrier for Bcl6-dependent Tfh differentiation, as it directly represses Bcl6 transcription and concurrently facilitates Blimp-1 expression [26]. Conversely, Blimp-1 is also critical for the acquisition of a full suppressive phenotype of Tfr cells, as Blimp-1–deficient Tfr cells exhibit impaired GC homing and reduced IL-10 and CTLA-4 expression [27, 28]. Additional studies revealed that the in vivo Tfr cell pool consists of both CD25^+^ and CD25^–^ cell populations, reflecting their differentiation status and positioning within the GC, where declining IL-2 concentrations facilitate CD25 downregulation and further Bcl6 upregulation to stabilize the mature Tfr phenotype [29, 30]. Although IL-21 and IL-6 redundantly promote Tfh cell differentiation via Stat3 [31, 32], IL-21 signaling constrains Tfr expansion, as *Il21r*^*–/–*^ mice display enhanced proliferation and increased CXCR5^hi^FoxP3^+^Bcl6^+^ Tfr cells after T-dependent immunization, underscoring the antagonistic role of IL-21 in Tfr differentiation [31]. Interestingly, FoxP3 expression can be induced in end-stage GC Tfh cells, potentially through the downregulation of IL-21, thereby contributing to the contraction of the GC [33]. We have recently shown that TGF-β together with IL-6 induces the expression of both CXCR5 and Bcl6 in Tfh cells in vitro [34]. TGF-β is also indispensable for peripheral Treg conversion into CXCR5^+^FoxP3^+^ Tfr cells, where TGF-βR signaling stabilizes FoxP3 expression and upregulates the expression of follicular homing receptors, and blockade of TGF-β in Tregs abrogates Nrp1^+^ Tfr cell generation in vivo [35]. Therefore, TGF-β plays a key role in shaping the GC environment and supports the functional specialization of both Tfh and Tfr cells.

While significant progress has been made in understanding Tfr biology in vivo, studying their function in vitro has remained challenging because of the lack of robust differentiation protocols that yield phenotypical and functional Tfr cells. Here, we describe a novel in vitro differentiation protocol that generates mouse Tfr-like cells with phenotypic, transcriptional, and functional properties closely resembling those of their in vivo counterparts. Using this model, we established that TGF-β is sufficient to induce iTfr cells from naïve CD4^+^ precursors to suppress Tfh cell-mediated class switching of B cells in vitro. Moreover, we determined the tight regulation of Tfr cell identity through TGF-β and IL-2 and identified the transcription factor c-Maf as a key determinant of the expression of the follicular homing receptor CXCR5 in Tfr cells.

## Results

### TGF-β induces FoxP3 and CXCR5 protein expression in Tfr cells in vitro

**Fig. 1:**
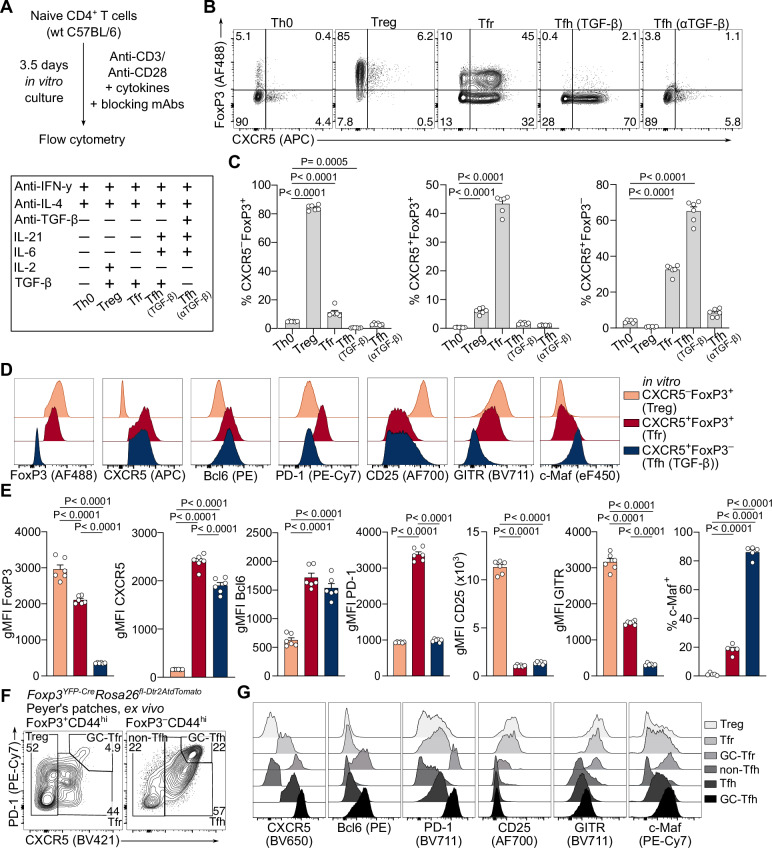
TGF-β induces CXCR5 and FoxP3 protein expression in murine Tfr-like cells in vitro. **A** Experimental outline: Naïve CD4^+^ T cells isolated from wildtype C57BL/6 mice were cultured in vitro in anti-CD3/CD28-coated 96-well flat-bottom cell culture plates for 3.5 days under the indicated T helper cell differentiation conditions. wt, wildtype; mAbs, monoclonal antibodies; IFN-γ, interferon-γ. **B** Representative flow cytometry contour plots of live CD4^+^ T cells stained for CXCR5 and FoxP3. **C** Quantification of frequencies of CXCR5^–^FoxP3^+^, CXCR5^+^FoxP3^+^, and CXCR5^+^FoxP3^–^ cells as gated in **B. D** Representative histograms of FoxP3, CXCR5, Bcl6, PD-1, CD25, GITR, and c-Maf expression in CXCR5^–^FoxP3^+^, CXCR5^+^FoxP3^+^, and CXCR5^+^FoxP3^–^ cells from Treg, Tfr, and Tfh (TGF-β) cell cultures, respectively. **E** Quantification of data in **D. F** Representative flow cytometry contour plots of live CD44^hi^FoxP3^+^ or CD44^hi^FoxP3^–^ CD4^+^ T cells from Peyer’s patches of *Foxp3*^*YFP-Cre*^.*Rosa26*^*fl-Dtr2AtdTomato*^ reporter mice stained for CXCR5 and PD-1. **G** Representative histograms of CXCR5, Bcl6, PD-1, CD25, GITR, and c-Maf expression in Treg, Tfr, GC-Tfr, non-Tfh, Tfh, and GC-Tfh cells as gated in **F**. Data in **B–E** and **F+G** are representative of more than five and two independent experiments, respectively, displaying mean ± SEM with *n* = 5 to 6 biological replicates per condition. Ordinary one-way ANOVA with Dunnett’s multiple comparisons test against Th0 **C** and Ordinary one-way ANOVA with Tukey’s multiple comparisons test **E**. Only significant *p* values < 0.05 are shown

**Fig. 2:**
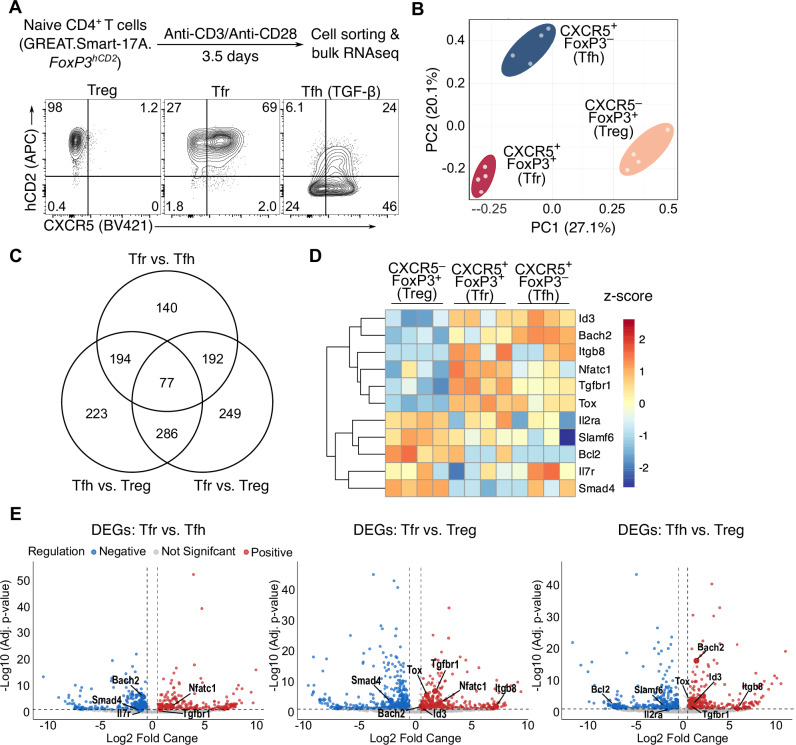
Transcriptomic signatures differentiate Treg, Tfr, and Tfh cells in vitro. **A** Experimental outline: Naïve CD4^+^ T cells from four different GREAT.Smart-17A.*FoxP3*^*hCD2*^ reporter mice were cultured in vitro for 3.5 days under Treg, Tfr, and Tfh (TGF-β) differentiation conditions. Live CD4^+^ CXCR5^–^FoxP3^+^, CXCR5^+^FoxP3^+^, and CXCR5^+^FoxP3^–^ cells were sorted from Treg, Tfr, and Tfh (TGF-β) cell cultures, respectively, for bulk RNA-seq. **B** PCA of the bulk RNA-seq data. **C** Euler diagram showing the overlap of differentially expressed genes among CXCR5^–^FoxP3^+^ Treg, CXCR5^+^FoxP3^+^ Tfr, and CXCR5^+^FoxP3^–^ Tfh (TGF-β) cells. **D** Heatmap of selected genes and their expression in sorted CXCR5^–^FoxP3^+^ Treg, CXCR5^+^FoxP3^+^ Tfr, and CXCR5^+^FoxP3^–^ Tfh (TGF-β) cells. Values represent rowwise z score-normalized expression across samples. **E** Volcano plots of differentially expressed genes (in blue and red) between CXCR5^–^FoxP3^+^ Treg, CXCR5^+^FoxP3^+^ Tfr, and CXCR5^+^FoxP3^–^ Tfh (TGF-β) cells

Naïve CD4⁺ T cells can upregulate FoxP3 expression in response to stimulation by TGF-β and TCR signaling, leading to the differentiation of induced regulatory T cells [36–38]. Similarly, while most Tfr cells are believed to originate from thymic-derived FoxP3⁺ Tregs [6, 23], some studies suggest that under strong TGF-β and TCR stimulation, FoxP3⁺CXCR5⁺ cells can arise from FoxP3⁻ precursors [20]. On this basis, we cultivated naïve wild-type C57BL/6 CD4⁺ T cells on plate-bound anti-CD3/anti-CD28 in the presence of TGF-β and blocking antibodies against IFN-γ as well as IL-4 to determine whether FoxP3 and CXCR5 protein coexpression could be induced in these cells (Fig. 1A). These cultures yielded more than 40% CXCR5^+^FoxP3^+^ double-positive cells (Fig. 1B, C and Supplementary Fig. 1A), indicating that the cells were iTfr cells [21]. Hereafter, these cultures were termed Tfr cell cultures. Minor populations of CXCR5^+^FoxP3^–^ and CXCR5^–^FoxP3^+^ cells, similar to TGF-β-induced Tfh cells and Treg cells, respectively, were also present in these cultures (Fig. 1B, C). Under Treg culture conditions, IL-2 enhanced TGF-β-mediated FoxP3^+^ induction while suppressing CXCR5 expression (Fig. 1B, C). As previously described [34], TGF-β together with IL-6 and IL-21 induced Tfh cells in Tfh (TGF-β) cell cultures, with almost no FoxP3^+^ cells present in these cultures (Fig. 1B, C). Th0 cell cultures and Tfh-like culture conditions, in which TGF-β signaling was blocked with anti-TGF-β antibodies (Tfh anti-TGF-β) [39], yielded very few CXCR5^+^ and/or FoxP3^+^ cells (Fig. 1B, C). Since Tfr cells share phenotypic markers of both Treg and Tfh cells, we next quantified the expression levels of various Tfr cell-associated markers in CXCR5^–^FoxP3^+^ Tregs, CXCR5^+^FoxP3^+^ Tfr cells, and CXCR5^+^FoxP3^–^ Tfh (TGF-β) cells derived from the respective cell cultures (Fig. 1D, E). CXCR5^+^FoxP3^+^ Tfr cells were Bcl6^high^PD-1^high^CD25^low^GITR^+^ (Fig. 1D, E), similar to their in vivo counterparts [29] in *Foxp3*^*YFP-Cre*^. *Rosa26*^*fl-Dtr2AtdTomato*^ reporter mice (Fig. 1F, G and Supplementary Fig. 1B and Supplementary Fig. 2). The transcription factor c-Maf, which is required for the generation of Tfh cells in vivo and Tfh (TGF-β) cells in vitro [34, 40–42], was also expressed in in vitro-differentiated Tfr cells (Fig. 1D, E) as well as in Tfr cells residing in Peyer’s patches (Fig. 1G and Supplementary Fig. 2). To assess the identity of in vitro-generated Tfr cells at the global transcriptional level, we cultured naïve CD4^+^ T cells from GREAT.Smart-17A.*FoxP3*^*hCD2*^ triple-reporter mice under Treg, Tfr, and Tfh (TGF-β) conditions (Fig. 2A). After 3.5 days, CXCR5^+^FoxP3^+^ Tfr, CXCR5^–^FoxP3^+^ Treg, and CXCR5^+^FoxP3^–^ Tfh cells were purified by cell sorting and subjected to bulk RNA-seq. A full overview of the gene expression values and DEG statistics is provided in Supplementary Data 1. Principal component analysis (PCA) revealed clear separation of the Treg, Tfr, and Tfh (TGF-β) cell populations (Fig. 2B). Euler display visualization revealed distinct overlapping and separate differentially expressed genes (DEGs) among the three cell populations (Fig. 2C). Reflecting the protein expression analyses, compared with Tfr and Tfh (TGF-β) cells, Tregs displayed higher levels of Il2ra (CD25) expression (Fig. 2D, E). They also expressed high levels of *Bcl2*, *Slamf6*, and *Smad4* (Fig. 2D, E). Tfr cells expressed particularly high levels of *Nfatc1*, which has been previously shown to regulate CXCR5 expression in Tfr cells [24]; *Tox*; as well as *Itgb8* and *Tgfbr1*, which are important for the function of TGF-β (Fig. 2D, E). In contrast, Id3 and Bach2 transcripts were enriched in Tfh (TGF-β) cells (Fig. 2D, E). In summary, these protein and gene expression data supported the distinct identity of the in vitro-generated Tfr cells in comparison to the related Treg and Tfh cell populations.

### In vitro-generated Tfr cells are functional

**Fig. 3:**
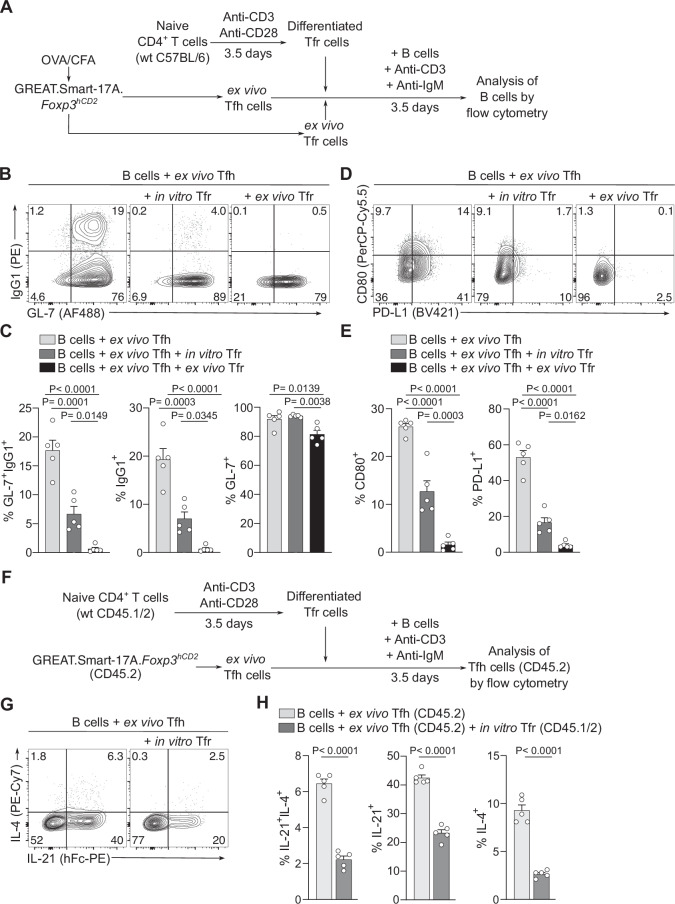
In vitro*-*generated Tfr cells are functional suppressors of B cells. **A** Experimental outline: Naïve CD4^+^ T cells from wildtype C57BL/6 mice were differentiated in vitro toward Tfr cells for 3.5 days. In parallel, live polyclonal CD19^−^CD8a^−^CD4^+^ FoxP3^−^CXCR5^+^PD-1^+^ Tfh and CD19^−^CD8a^−^CD4^+^FoxP3^+^CXCR5^+^ Tfr cells were sorted from inguinal and axillary lymph nodes of GREAT.Smart-17A.*FoxP3*^*hCD2*^ reporter mice immunized subcutaneously with OVA/CFA 7 days earlier and tested together with the in vitro–generated Tfr cell population in cocultures with purified wildtype C57BL/6 B cells. After 3.5 days, the cultured B cells were analyzed by flow cytometry. **B** Flow cytometry contour plots showing the frequency of GL-7^+^IgG1^+^ B cells after 3.5 days of coculture with ex vivo-sorted Tfh cells alone or together with in vitro-differentiated Tfr or ex vivo-sorted Tfr cell populations. Gated on live CD19^+^CD4^–^ cells. **C** Quantification of data in **B. D** Flow cytometry contour plots showing the frequencies of CD80^+^ and PD-L1^+^ cells among live CD19^+^CD4^–^ B cells after 3.5 days of coculture. **E** Quantification of the data in **D**. **F** Experimental outline. **G** Flow cytometry contour plots showing the frequency of IL-21^+^ and IL-4^+^ cells among ex vivo-sorted Tfh cells from unimmunized GREAT.Smart-17A.*FoxP3*^*hCD2*^ reporter mice (CD45.2^+^CD45.1^−^) after 3.5 days of coculture with in vitro-differentiated (CD45.2^+^CD45.1^+^) Tfr cell populations. Gated on live CD19^–^CD4^+^CD45.2^+^CD45.1^−^ cells. **H** Quantification of the data in **F**. Data in **B–E** and **G+H** are representative of three and two individual experiments, respectively, displaying mean ± SEM with *n* = 4–5 biological replicates per condition. Ordinary one-way ANOVA with Tukey’s multiple comparisons test C+E and unpaired t test H. Only significant *p* values < 0.05 are shown

Since Tfr cells critically control Tfh cell-mediated B-cell functions [43], we next compared the suppressive capacity of in vitro-generated Tfr cells and in vivo-generated Tfr cells (Fig. 3A). To this end, Tfr cell cultures or sorted FoxP3^+^CXCR5^+^ cells from GREAT.Smart-17A.*FoxP3*^*hCD2*^ reporter mice that had previously been immunized with OVA/CFA were cocultured together with ex vivo-sorted polyclonal Tfh cells and B cells in the presence of anti-CD3/anti-IgM, similar to previous studies [23]. Importantly, in vitro-generated Tfr cells potently suppressed class-switch recombination to IgG1, as did in vivo-generated Tfr cells (Fig. 3B, C). Although in vitro-generated Tfr cells did not reduce the expression of the activation marker GL-7 on B cells, the expression levels of both the costimulatory molecules PD-L1 and CD80 were significantly decreased on B cells (Fig. 3D, E). In addition, analysis of T–B-cell cocultures with ex vivo-sorted Tfh cells further revealed reduced IL-21 and IL-4 production by Tfh cells when these cells were cocultured together with in vitro-generated Tfr cells (Fig. 3F–H). Taken together, these data highlight the functional ability of in vitro-generated Tfr cells to effectively inhibit Tfh-B-cell interactions and class switching.

### Balanced TGF-β and IL-2 signaling shape Tfr cell induction and maintenance in vitro

**Fig. 4:**
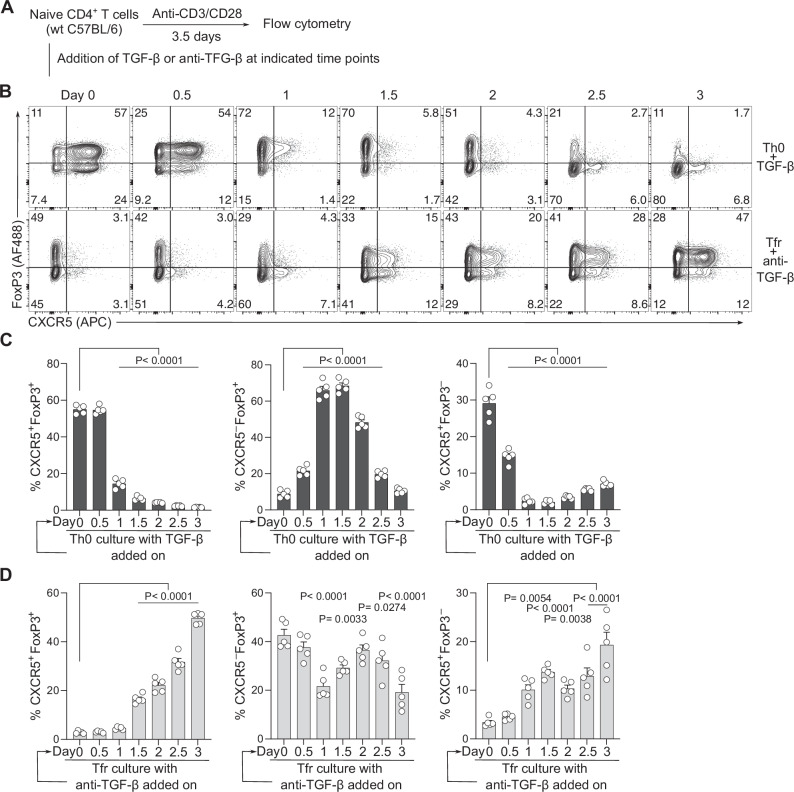
TGF-β is essential for the early stages of Tfr cell differentiation. **A** Experimental outline: Addition of TGF-β to Th0 cell cultures or anti-TGF-β to Tfr cell cultures at the indicated time points. **B** Representative flow cytometry contour plots gated on live CD4^+^ T cells stained for CXCR5 and FoxP3. **C+D** Quantification of the frequencies of CXCR5^–^FoxP3^+^, CXCR5^+^FoxP3^+^, and CXCR5^+^FoxP3^–^ cells after 3.5 days of in vitro Th0 and Tfr cell cultures with 5 ng/ml TGF-β or 10 µg/ml anti-TGF-β added at indicated time points, respectively. Data are representative of three independent experiments displaying mean ± SEM with *n* = 5 biological replicates per condition. Ordinary one-way ANOVA with Dunnett’s multiple comparisons test against day 0. Only significant *p* values < 0.05 are shown

Next, we investigated the requirements and kinetics of efficient FoxP3 and CXCR5 induction in vitro by either adding TGF-β to Th0 cultures or blocking them in Tfr cultures at different time points following naïve wild-type C57BL/6 CD4^+^ T-cell activation (Fig. 4A). We observed a progressive decrease in the frequency of CXCR5⁺FoxP3⁺ Tfr cells by day 3.5 of culture when TGF-β was added to Th0 (anti-IFN-γ, anti-IL-4) cells at later time points (Fig. 4B, C). Interestingly, the early presence of TGF-β clearly favored the development of CXCR5⁺FoxP3⁺ cells over that of CXCR5^–^FoxP3⁺ cells (Fig. 4B, C and Supplementary Fig. 3A). In contrast, the addition of TGF-β to Th0 cultures during the time window from day 1 to approximately day 2.5 strongly induced FoxP3 single-positive Treg cells (Fig. 4B, C and Supplementary Fig. 3A), with almost no possibility of inducing CXCR5 expression in any of the CD4^+^ T cells (Fig. 4B, C and Supplementary Fig. 3A). CXCR5⁺FoxP3^–^ cells displayed mostly similar developmental kinetics to those of CXCR5⁺FoxP3⁺ cells (Fig. 4B, C and Supplementary Fig. 3A) and to those previously described for Tfh (TGF-β) cells [34]. In a complementary approach, neutralization of TGF-β in the Tfr cell culture condition (anti-IFN-γ, anti-IL-4, and anti-TGF-β) resulted in a significant reduction in the percentage of CXCR5⁺FoxP3⁺ cells by day 3.5, especially when TGF-β was neutralized early during the culture (Fig. 4B, D and Supplementary Fig. 3B). The sharp decrease in the CXCR5⁺FoxP3⁺ cell population upon anti-TGF-β treatment was accompanied by reduced expression of Bcl6 and c-Maf and an increase in the frequency of CD25^+^ cells (Supplementary Fig. 3C, D). Together, these results suggested that TGF-β was required for the induction of CXCR5⁺FoxP3⁺ Tfr cells.

**Fig. 5:**
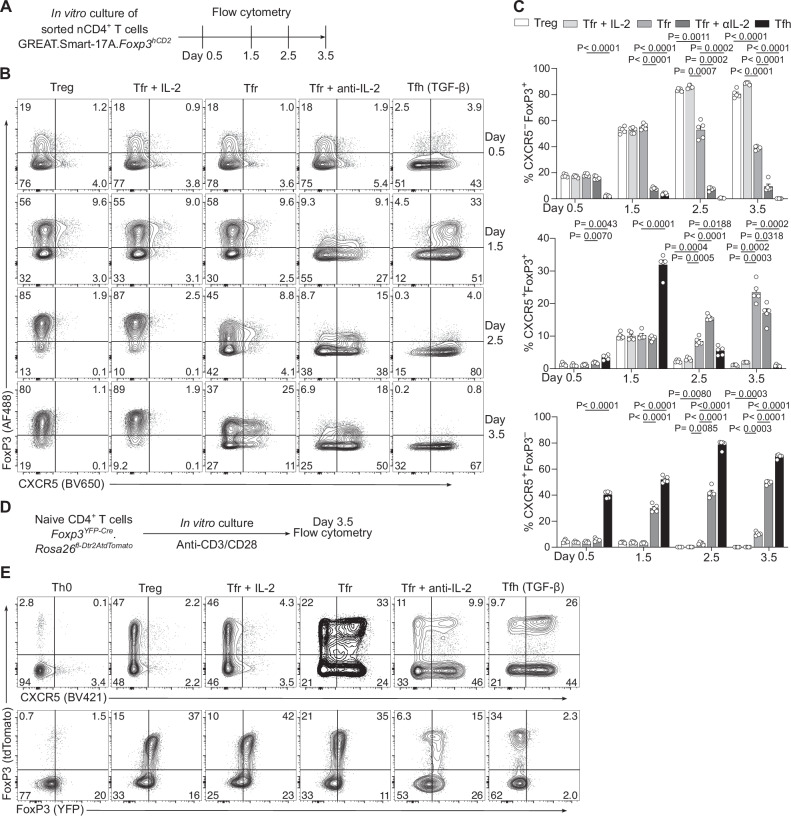
Stepwise differentiation of Tfr cells from FoxP3^+^ precursors. **A** Experimental outline: Naïve CD4^+^ T cells were sorted from GREAT.Smart-17 A.*FoxP3*^*hCD2*^ triple-reporter mice and cultured under the indicated T helper cell differentiation conditions in vitro. At the indicated time points, cells were analyzed by flow cytometry. **B** Representative flow cytometry contour plots gated on live CD4^+^ T cells stained for CXCR5 and FoxP3 after 0.5, 1.5, 2.5, and 3.5 days of in vitro Treg, Tfr, or Tfh (TGF-β) culture. Where indicated, 40 ng/ml IL-2 or 10 µg/ml anti-IL-2 were added to the Tfr culture. **C** Quantification of the frequencies of CXCR5^–^FoxP3^+^, CXCR5^+^FoxP3^+^, and CXCR5^+^FoxP3^–^ cells in Treg, Tfr, or Tfh (TGF-β) cultures. **D** Experimental outline. **E** Representative flow cytometry contour plots gated on live CD4^+^ T cells displaying tdTomato and YFP expression after 3.5 days of in vitro culture under Th0, Treg, Tfr, or Tfh (TGF-β)-polarizing conditions. Where indicated, 40 ng/ml IL-2 or 10 µg/ml anti-IL-2 were added to the Tfr cell culture. Data are representative of four and two independent experiments, respectively with 5 **B+C** or 2 **D+E** biological replicates per condition. **C** shows mean ± SEM. Two-way ANOVA with Dunnett’s multiple comparisons test against the Tfr condition. Only significant *p* values < 0.05 are shown

The unique differentiation process of Tfr cells resembles a paradox: Tfr cells reconcile the seemingly opposing cytokine signals of TGF-β, which induces CXCR5, and IL-2, which stabilizes FoxP3 but inhibits CXCR5 expression [44–46]. When the dynamics of FoxP3 and CXCR5 expression in Treg, Tfr, and Tfh (TGF-β) cell cultures were examined over a 3.5-day time course (Fig. 5A), FoxP3^+^ cells started to emerge on day 0.5 under Treg and Tfr conditions, with robust CXCR5 expression being restricted to Tfh (TGF-β) conditions at this time (Fig. 5B, C and Supplementary Fig. 4A). One day later, approximately 60% of the CXCR5^–^FoxP3⁺ cells were present in the Treg and Tfr conditions, and approximately 10% of the CXCR5^+^FoxP3⁺ cells started to develop from the pool of FoxP3^+^ cells, with only a few CXCR5^+^FoxP3^–^ cells present at this stage (Fig. 5B, C). Under Tfh (TGF-β) cell-polarizing conditions, approximately 50% of the CXCR5^+^FoxP3^–^ cells were present on day 1.5 (Fig. 5B, C and Supplementary Fig. 4A). In addition, 30% of the Tfh (TGF-β)-cultured cells coexpressed CXCR5 and FoxP3, which likely represents the result of T-cell activation, as few CXCR5^–^FoxP3⁺ cells were present at this stage, and almost all FoxP3-expressing cells were completely diminished by day 3.5 of culture (Fig. 5B, C and Supplementary Fig. 4A). This was also reflected in the slightly longer presence of the huCD2 FoxP3 reporter in the Tfh (TGF-β) condition (Supplementary Fig. 5A, B) than in direct intracellular FoxP3 staining (Fig. 5B). In contrast, the development of the CXCR5^+^FoxP3⁺ fraction continued to occur under the Tfr cell culture condition by day 3.5 (Fig. 5B, C and Supplementary Fig. 4A and Supplementary Fig. 5A, B). The addition of IL-2 to the Tfr cells significantly reduced the frequencies of CXCR5⁺FoxP3⁺ and CXCR5^+^FoxP3^–^ cells from day 2.5 onward, whereas it increased the frequency of CXCR5^–^FoxP3⁺ cells (Fig. 5B,C and Supplementary Fig. 4A and Supplementary Fig. 5A,B). In contrast, the neutralization of IL-2 signaling under Tfr conditions resulted in higher CXCR5^+^FoxP3^–^ and lower CXCR5^–^FoxP3⁺ cell frequencies (Fig. 5B, C and Supplementary Fig. 4A and Supplementary Fig. 5A, B). Finally, CD25 expression increased upon IL-2 supplementation but decreased following anti-IL-2 treatment, whereas c-Maf expression was reciprocally regulated (Supplementary Fig. 4B, C). Since these data supported a model in which CXCR5^+^FoxP3⁺ Tfr cells were derived from Foxp3^+^ precursors and in which their development was shaped by the balanced action of TGF-β and IL-2, we next used a fate-mapping approach with *Foxp3*^*YFP-Cre*^. *Rosa26*^*fl-Dtr2AtdTomato*^ reporter mice, in which *Rosa26*^*fl-Dtr2AdtTomato*^ reporter alleles were irreversibly induced upon *Foxp3*-driven Cre recombinase activity, to further elucidate the ontogeny of CXCR5^+^FoxP3⁺ Tfr cells (Fig. 5D). Under Tfr cell culture conditions, all three CXCR5^–^FoxP3⁺, CXCR5^+^FoxP3^+^, and CXCR5^+^FoxP3^–^ cell populations were identified by tdTomato and CXCR5 expression (Fig. 5E). More than half of the tdTomato^+^ cells coexpressed YFP, indicating active *Foxp3* expression (Fig. 5E). As controls, Th0 cultures did not show much tdTomato or YFP expression, whereas Treg cultures yielded approximately 50% tdTomato and YFP-positive cells but almost no CXCR5-expressing cells (Fig. 5E). The addition of IL-2 to the Tfr cells effectively reduced CXCR5 expression and shifted the split YFP expression among the tdTomato^+^ cells in the Tfr condition to YFP^+^ Treg cells in the Tfr+IL-2 condition (Fig. 5E). In contrast, the addition of anti-IL-2 antibodies reduced the frequencies of tdTomato- and YFP-positive cells in the Tfr cell culture, simultaneously increasing the Foxp3^–^CXCR5^+^ fraction (Fig. 5E). The tdTomato-fate-mapping approach also revealed that most of the tdTomato^+^ cells in the Tfh (TGF-β) condition were CXCR5^+^, with some coexpressing tdTomato, but no cells in this condition actively expressed FoxP3 (YFP) at the time of analysis on day 3.5 of culture (Fig. 5E); thus, the data from the time-course experiments were reconstructed (Fig. 5E).

### Molecular requirements for Tfr cell generation in vitro

**Fig. 6:**
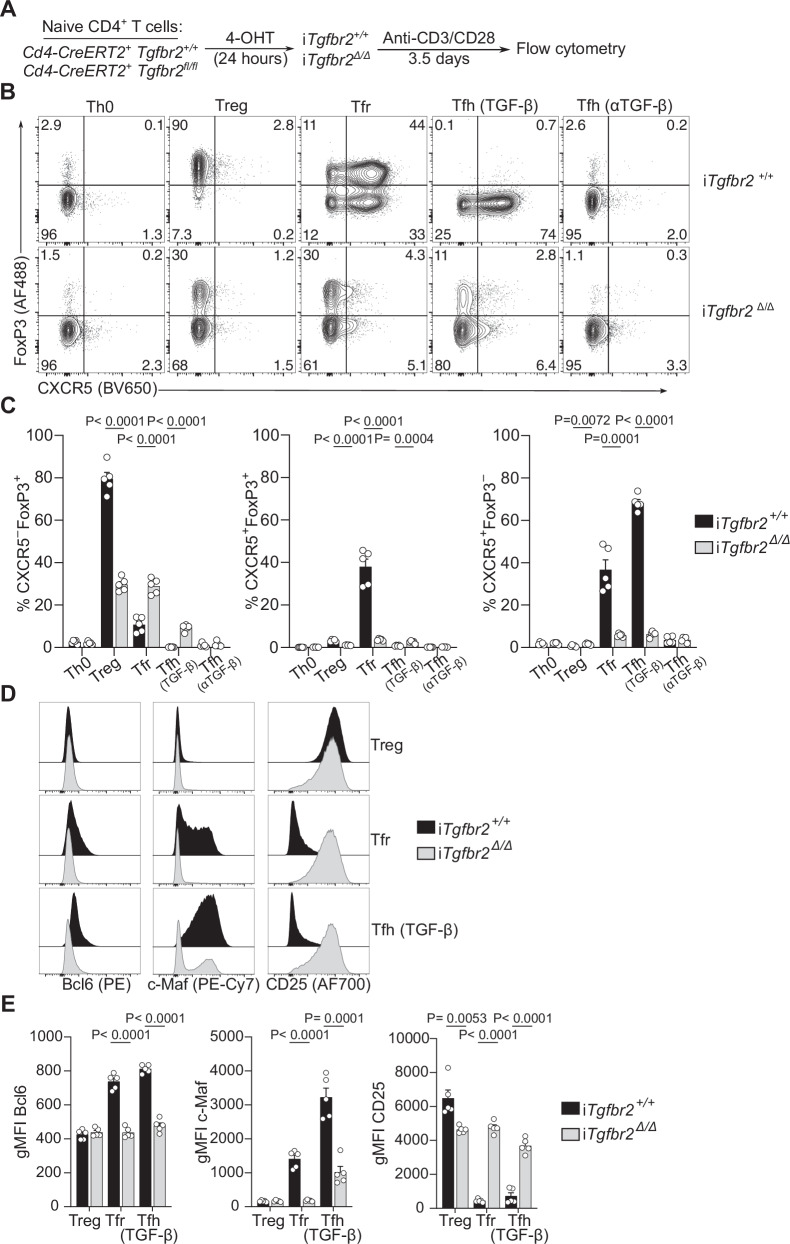
TGF-β signaling is required for Tfr cell generation in vitro. **A** Experimental outline: Naïve *Cd4*-*CreERT2*^*+*^ *Tgfbr2*^*+/+*^ *Rosa26*^*fl-Stop-fl-YFP*^ (*iTgfbr2*^*+/+*^) and *Cd4*-*CreERT2*^*+*^ *Tgfbr2*^*fl/fl*^ *Rosa26*^*fl-Stop-fl-YFP*^ (*iTgfbr2*^*Δ/Δ*^) CD4^+^ T cells were treated with 4-hydroxytamoxifen (4-OHT) for 24 h and then differentiated under T helper cell polarizing conditions for 3.5 days in vitro **B** Representative flow cytometry contour plots gated on live CD4^+^ T cells and displaying CXCR5 and FoxP3 expression. **C** Quantification of the frequencies of CXCR5^–^FoxP3^+^, CXCR5^+^FoxP3^+^, and CXCR5^+^FoxP3^–^ cells among live CD4^+^ T cells. **D** Representative histograms of the gMFI of Bcl6, c-Maf, and CD25 in live CD4^+^ T cells from Treg, Tfr, and Tfh (TGF-β) cell cultures. **E** Quantification of the data in **D**. The data are representative of four independent experiments and are presented as the mean ± SEM, with *n* = 5 biological replicates per condition. Multiple unpaired t tests were used. Only significant *p* values < 0.05 are shown

Having established that TGF-β is a critical cytokine for the induction of in vitro Tfr cells, we next evaluated the impact of CD4^+^ T-cell-intrinsic disruption of TGF-β signaling (Fig. 6A). To this end, tamoxifen-treated *Cd4*-*CreERT2*^*+*^ *Tgfbr2*^*+/+*^ (i*Tgfbr2*^*+/+*^) and *Cd4*-*CreERT2*^*+*^ *Tgfbr2*^*fl/fl*^ (i*Tgfbr2*^*Δ/Δ*^) CD4^+^ T cells were cultured under different T helper cell differentiation conditions (Fig. 6B). Compared with i*Tgfbr2*^*+/+*^ control cells, i*Tgfbr2*^*Δ/Δ*^ cells exhibited a significant reduction in the frequency of CXCR5^+^FoxP3^+^ Tfr cells, with a concomitant increase in the number of CXCR5^–^FoxP3^+^ cells (Fig. 6B, C). The effects of TGF-β ablation on CXCR5 expression under Tfh (TGF-β) conditions were similar to those in our previous report, with almost no CXCR5 expression detectable under i*Tgfbr2*^*Δ/Δ*^ Tfh (TGF-β) conditions [34] (Fig. 6B, C). Th0 and Tfh (anti-TGF-β) conditions did not induce CXCR5 [34] or FoxP3 expression, independent of *Tgfbr2* ablation (Fig. 6B, C). Interestingly, while i*Tgfbr2*^*Δ/Δ*^ Tfh (TGF-β) cells displayed an almost complete loss of CXCR5 expression, FoxP3 expression was induced in approximately 10–15% of the cells, which could be attributed to increased IL-2 production in these cultures [34] (Fig. 6B, C). The number of CXCR5^–^FoxP3^+^ cells in i*Tgfbr2*^*Δ/Δ*^ Treg cultures was reduced to levels similar to those observed in i*Tgfbr2*^*Δ/Δ*^ Tfr cell cultures (Fig. 6B, C). Quantitative analysis of Tfr-associated markers in Treg, Tfr, and Tfh (TGF-β) cell cultures revealed a strongly increased gMFI of CD25 in i*Tgfbr2*^*Δ/Δ*^ cells cultured under Tfr and Tfh conditions and a slight reduction in CD25 expression in i*Tgfbr2*^*Δ/Δ*^ Treg cultures (Fig. 6D, E). The expression levels of both Bcl6 and c-Maf were significantly reduced in Tfr and Tfh (TGF-β) cells from i*Tgfbr2*^*Δ/Δ*^ cells, suggesting that TGF-β signaling is not only essential for the generation of Tfr cells but also plays a critical role in regulating the expression of key transcription factors and costimulatory molecules that define the identity of Tfr cells, which are similar to Tfh cells [34].

**Fig. 7:**
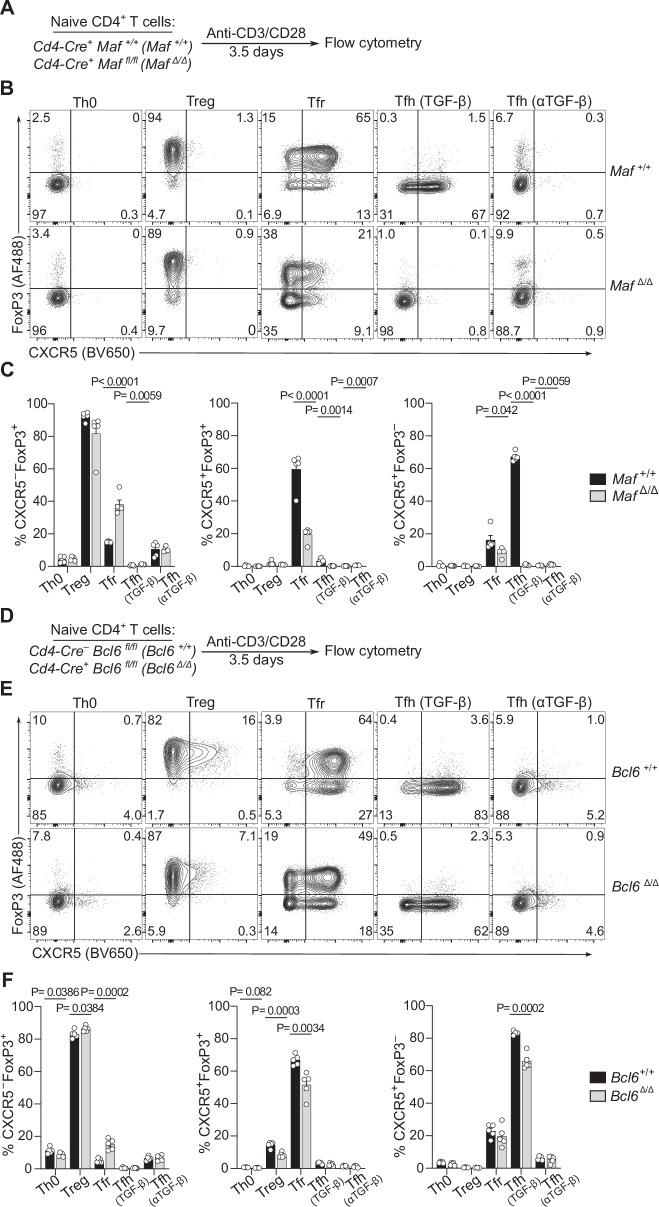
c-Maf, but not Bcl6, is required for robust CXCR5 expression in murine Tfr-like cells in vitro. **A** Experimental outline: Naïve *Cd4*-*Cre*^*+*^ *Maf* ^*+/+*^ (*Maf* ^*+/+*^) and *Cd4*-*Cre*^*+*^ *Maf* ^*fl/fl*^ (*Maf* ^*Δ/Δ*^) CD4^+^ T cells were cultured under different T helper cell differentiation conditions for 3.5 days. **B** Representative flow cytometry contour plots gated on live CD4^+^ T cells stained for CXCR5 and FoxP3. **C** Quantification of the frequencies of CXCR5^–^FoxP3^+^, CXCR5^+^FoxP3^+^, and CXCR5^+^FoxP3^–^ cells among live CD4^+^ T cells, as gated in **B**. **D** Experimental outline: Naïve *Cd4*-*Cre*^–^ *Bcl6* ^*fl/fl*^ (Bcl6 ^*+/+*^) and *Cd4*-*Cre*^*+*^ *Bcl6* ^*fl/fl*^ (*Bcl6* ^*Δ/Δ*^) CD4^+^ T cells were cultured under different T helper cell differentiation conditions for 3.5 days. **E** Representative flow cytometry contour plots gated on live CD4^+^ T cells stained for CXCR5 and FoxP3. **F** Quantification of the frequencies of CXCR5^–^FoxP3^+^, CXCR5^+^FoxP3^+^, and CXCR5^+^FoxP3^–^ cells among live CD4^+^ T cells, as gated in **E**. Data in **B+C** and **E+F** are representative of four and two independent experiments, respectively, displaying mean ± SEM with *n* = 5 biological replicates per condition. Multiple unpaired t tests were performed and only significant *p* values < 0.05 are shown

Since c-Maf is known to drive CXCR5 expression in Tfh and Tfr cells [34, 41, 42, 47], c-Maf likely contributes to the regulation of CXCR5 expression in Tfr cells in vitro. Hence, we tested the ability of naïve CD4^+^ T cells from *Cd4-Cre*^*+*^ *Maf*^*fl/fl*^ (*Maf*^*Δ/Δ*^) mice to differentiate into Tfr cells in vitro (Fig. 7A). c-Maf-deficient T cells were significantly impaired in the generation of CXCR5^+^FoxP3^+^ and CXCR5^+^FoxP3^–^ cells under Tfr cell conditions, while the percentage of CXCR5^–^FoxP3^+^ cells significantly increased (Fig. 7B, C). CXCR5 expression was not strongly downregulated in the Tfr cells compared with that in the Tfh cells, in which no CXCR5 expression was detected in the *Maf*^*Δ/Δ*^ cells, as previously described [34] (Fig. 7B, C). *Maf* deficiency did not affect the frequency of CXCR5^–^FoxP3^+^ cells under Treg conditions (Fig. 7B, C). Consistent with previous reports, *Maf* deficiency resulted in reduced frequencies and numbers of Tfh and Tfr cells in vivo [34, 42, 47] (Supplementary Fig. 6A, B). A similar trend was observed following subcutaneous immunization with OVA/CFA, in which T-cell-specific *Maf*-deficient mice also displayed reduced frequencies of Tfh and Tfr cells in the draining lymph nodes (Supplementary Fig. 6C–E).

While it is widely known that Bcl6 is required for Tfh cell differentiation in vivo [48–50], early initiation of the Tfh program – including CXCR5 expression – can also occur through Bcl6-independent mechanisms, e.g., through Ascl2, which functions upstream of Bcl6 [51]. Furthermore, CXCR5 expression in Tfr cells requires NFAT2 [24], whose expression was also upregulated in our in vitro Tfr cell cultures (Fig. 2D). We therefore tested the ability of *Bcl6*-deficient naïve CD4^+^ T cells to differentiate into Tfr cells in vitro (Fig. 7D). Bcl6 expression was not essentially required for the in vitro differentiation of Tfh (TGF-β) cells [34], in contrast to the mesenteric lymph nodes of *Bcl6*^*Δ/Δ*^ mice in vivo, in which we observed a marked reduction in the GC-Tfh cell frequency (Fig. 7E, F and Supplementary Fig. 6F, G). Similarly, the frequency of *Bcl6*^*Δ/Δ*^ CD4^+^ T cells decreased slightly but was still substantial among CXCR5^+^FoxP3^+^ Tfr and CXCR5^+^FoxP3^–^ Tfh cells, whereas the frequency of CXCR5^–^FoxP3^+^ Treg cells slightly increased in vitro (Fig. 7E, F). In line with previous reports [6, 12, 52], in vivo GC-Tfr frequencies were significantly reduced in *Bcl6*^*Δ/Δ*^ mice (Supplementary Fig. 6F, G). Although we did not observe a strong effect of *Bcl6*-deficiency on Tfh and Tfr cell differentiation in vitro, compared with *Maf*-deficiency, Bcl6 could still be important for their function. Thus, to determine whether Bcl6 was required for Tfh-mediated B-cell help, we next evaluated the capacity of in vitro-differentiated *Bcl6*^*+/+*^ and *Bcl6*-deficient (*Bcl6*^*Δ/Δ*^) Tfh (TGF-β) cells to support B-cell activation and class-switching to IgG1 in a T–B-cell coculture assay (Supplementary Fig. 7A). While *Bcl6*-deficient Tfh (TGF-β) cells still supported B-cell activation, IgG1 class-switching was significantly reduced (Supplementary Fig. 7B, C). Cocultures of in vitro-differentiated *Bcl6*^*+/+*^ Tfh (TGF-β) cells with *Bcl6*^*Δ/Δ*^ Tfr cells resulted in a significant increase in IgG1-producing and GL-7-expressing B cells compared with cocultures with *Bcl6*^*+/+*^ Tfr cells (Supplementary Fig. 7D–F). These results suggest that early Tfh/Tfr cell differentiation can occur in a largely Bcl6-independent manner in vitro but that Bcl6 remains important for optimal Tfh and Tfr cell function.

### Stability and plasticity of in vitro-generated Tfr cells

**Fig. 8:**
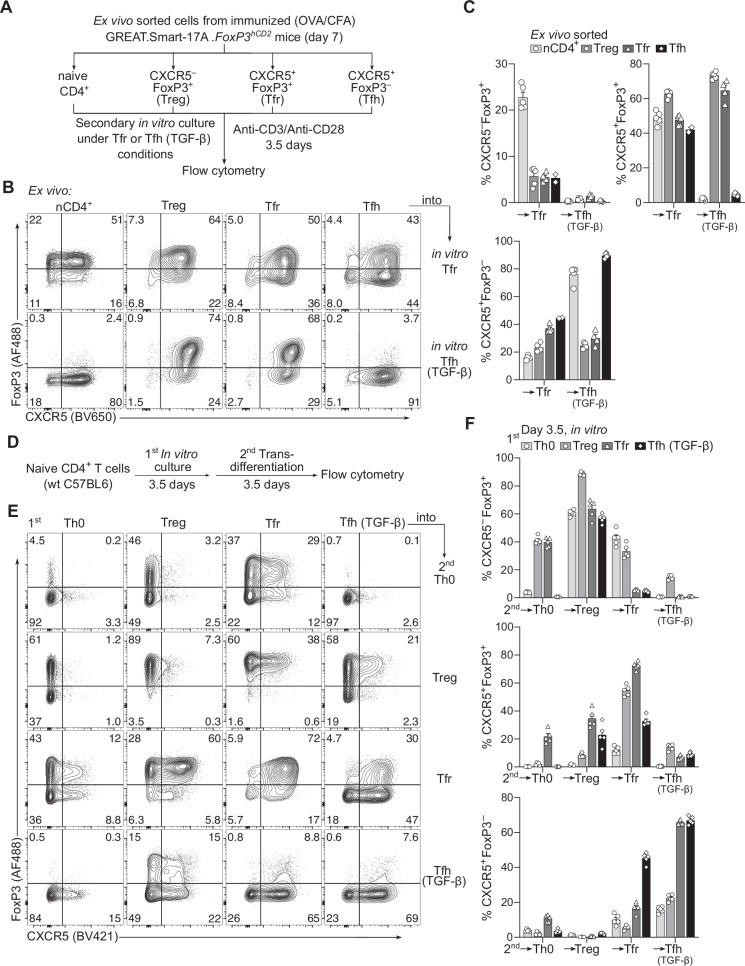
Stability and plasticity of Treg, Tfr, and Tfh cells. **A** Experimental outline: GREAT.Smart-17A.*FoxP3*^*hCD2*^ triple-reporter mice were immunized subcutaneously with OVA/CFA and 7 days later, live naïve CD4^+^ T cells as well as CXCR5^–^FoxP3^+^ Tregs, CXCR5^+^FoxP3^+^ Tfrs, and CXCR5^+^FoxP3^–^ Tfh cells were sorted and further cultivated in vitro in anti-CD3/CD28-coated cell culture plates for 3.5 days under Tfr or Tfh (TGF-β) polarizing conditions. **B** Representative flow cytometry contour plots gated on live CD4^+^ T cells stained for CXCR5 and FoxP3. **C** Quantification of the data in **B**. **D** Experimental outline: Naïve CD4^+^ T cells from wildtype C57BL/6 mice were cultured under Th0, Treg, Tfr, or Tfh (TGF-β)-polarizing conditions. After 3.5 days, cells were split 1:8, replated on fresh anti-CD3/CD28-coated cell culture plates, and either maintained in their original culture condition with fresh medium or trans-differentiated under secondary Th0, Treg, Tfr, or Tfh (TGF-β)-polarizing conditions for another 3.5 days. **E** Representative flow cytometry contour plots gated on live CD4^+^ T cells stained for CXCR5 and FoxP3. **F** Quantification of the data in **E**. Data in **B+C** and **E+F** are each representative of three independent experiments, displaying mean ± SEM with *n* = 2-5 biological replicates per condition

T helper cell plasticity enables rapid adaptation to changing microenvironments [53]. Initial in vitro work indicated that Tfh cells resist conversion toward a FoxP3^+^ phenotype [8]; however, subsequent studies have shown that low-dose IL-2 therapy can convert Tfh cells into Tfr cells in SLE patients [54] and that late-stage GC Tfh cells can acquire FoxP3 expression, potentially contributing to GC contraction [33]. To assess the conversion potential of T helper cell subsets in vitro, GREAT.Smart-17A.*Foxp3*^*hCD2*^ triple-reporter mice were immunized subcutaneously with OVA/CFA, and 7 days later, Treg, Tfr, and Tfh cells were sorted by flow cytometry from skin-draining lymph nodes (Fig. 8A). These ex vivo-isolated T helper cells were then stimulated on plate-bound anti-CD3/anti-CD28 antibodies under Tfr- or Tfh-polarizing conditions, with a separate set of naïve CD4^+^ T cells that were cultured under the same conditions in parallel (Fig. 8A). Under secondary in vitro Tfr-polarizing conditions, all the sorted populations acquired a CXCR5^+^FoxP3^+^ Tfr cell population (Fig. 8B, C and Supplementary Fig. 1C and Supplementary Fig. 8A, B). IL-6, IL-21 and TGF-β induced mainly a CXCR5^+^FoxP3^–^ Tfh cell phenotype in naïve CD4^+^ T cells, and such a CXCR5^+^FoxP3^–^ Tfh phenotype was also maintained by ex vivo-sorted Tfh cells when they were cultured under the same Tfh (TGF-β)-polarizing conditions (Fig. 8B, C and Supplementary Fig. 8A, B). Strikingly, despite the presence of IL-6 and IL-21, two cytokines that normally suppress FoxP3 under Tfh-polarizing conditions (Fig. 1B, C), both ex vivo-sorted Treg cells and Tfr cells retained FoxP3 expression but strongly upregulated CXCR5 expression to generate CXCR5^+^FoxP3^+^ Tfr cells (Fig. 8B, C and Supplementary Fig. 8A, B). These data indicated that previously established FoxP3^+^ Treg cells can be transformed into Tfr cells in the presence of IL-6/IL-21 (Fig. 8B, C and Supplementary Fig. 8A, B). To further elucidate these trans-differentiation processes, we first established in vitro cultures with naïve CD4^+^ T-cell precursors from wild-type C57BL/6 mice to generate Th0, Treg, Tfr, and Tfh (TGF-β) cells (Fig. 8D). In the next step, these cells were purified from the cultures and replated under secondary Th0, Treg, Tfr, and Tfh (TGF-β)-polarizing conditions together with plate-bound anti-CD3/anti-CD28 stimulation (Fig. 8D). While TGF-β supplementation was necessary to maintain stable CXCR5 expression in the original Tfr and Tfh (TGF-β) cells replated under Treg, Tfr, and Tfh (TGF-β) conditions, CXCR5 expression by original Tfr and Tfh (TGF-β) cells was markedly reduced and completely absent, respectively, under Th0 replating conditions (Fig. 8E, F). In general, FoxP3 expression was stable in secondary cultures of the primary Treg and Tfr populations; even under Th0 culture conditions lacking supplemented TGF-β, a substantial number of the original Treg and Tfr cells remained FoxP3^+^ (Fig. 8E, F). Compared with their primary in vivo counterparts, primary in vitro-differentiated Treg and Tfr cells were more susceptible to the inhibitory effects of IL-6 and IL-21 on FoxP3 expression in secondary cultures (Fig. 8E, F) (Fig. 8B, C). Furthermore, the majority of the emerging CXCR5^+^ cells in these Treg-to-Tfh and Tfr-to-Tfh cell cultures were FoxP3-negative (Fig. 8E, F). Initially differentiated FoxP3^+^ Tregs gave rise to CXCR5^+^FoxP3^+^ cells under secondary Tfr cell culture conditions (Fig. 8E, F). Similarly, ex vivo-sorted Tregs from GREAT.Smart-17A.*Foxp3*^*hCD2*^ triple-reporter mice cultured under Treg-polarizing conditions gave rise to CXCR5^+^FoxP3^+^ cells, albeit at lower frequencies than ex vivo-sorted Tregs cultured under Tfr-polarizing conditions (Supplementary Fig. 8D, E). The addition of anti-IL-2 to the Treg-to-Tfr cell culture further reduced overall FoxP3 expression and particularly increased the frequency of CXCR5^+^FoxP3^–^ cells (Supplementary Fig. 8D, E). IL-2 in concert with TGF-β reduced the CXCR5^+^FoxP3^+^ phenotype in the initially cultured Tfr cells under secondary Treg conditions (Fig. 8E, F). In comparison to the Tfr-to-Tfr cultures, the Tfr-to-Treg cultures also largely lacked CXCR5^+^FoxP3^+^ cells but contained more CXCR5^–^FoxP3^+^ cells (Fig. 8E, F). Taken together, these findings established the identity and developmental pathways of Tfr cells in vitro and highlighted the ability to reprogram and reinforce regulatory follicular cell programs in T helper cell subsets.

## Discussion

In the present study, we established an in vitro protocol to generate Tfr cells derived from naïve CD4^+^ T-cell precursors as well as from established Tregs, thereby providing a platform to elucidate their differentiation pathways and suppressive functions. Although Tfr cells are thought to originate predominantly from thymus-derived Tregs [6–8], evidence suggests that they can also arise from naïve CD4^+^ T cells via peripheral induction following immunization, for example, in the presence of adjuvants such as incomplete Freund’s adjuvant (IFA) [20, 21]. A more recent study further challenged the previous dogma by providing evidence that human tonsillar Tfr cells can originate either from Tregs or from Tfh cells [55]. In our in vitro system, TGF-β was both necessary and sufficient to induce CXCR5^+^FoxP3^+^ Bcl6^hi^PD-1^hi^CD25^lo^GITR^int^ Tfr cells with the ability to suppress Tfh cell-induced class-switching to IgG1 in B cells, which phenotypically mimicked their in vivo counterparts. A loss of TGF-β signaling was associated with a significant reduction in the expression of key follicular T-cell markers, including CXCR5, Bcl6, and c-Maf, suggesting impaired acquisition of the follicular T-cell program in Tfr cells, similar to our previous findings in Tfh cells [34]. Conversely, abrogation of this pathway resulted in a concomitant increase in CD25 expression in both Tfr and Tfh cells, thereby enhancing their responsiveness to IL-2. This increased IL-2 sensitivity together with residual, low-level TGF-β pathway activity due to the experimental setup of tamoxifen-induced gene ablation may account for the induction of Foxp3 in *Tgfbr2*-deficient Tfh cell cultures and the maintenance of FoxP3 expression in *Tgfbr2*-deficient Tfr cell cultures. Furthermore, our data indicate that upon TCR stimulation, Tfr cells initially exhibit high levels of CD25 expression followed by upregulation of FoxP3 expression. The subsequent downregulation of CD25 coincides with the emergence of CXCR5^+^ cells from FoxP3^+^ precursors, accompanied by the expression of Bcl6. Notably, similar to their in vivo counterparts [29, 46], our in vitro-generated Tfr cells displayed a lower FoxP3 gMFI, a feature that has also been reported under IL-2–deprived conditions, which is consistent with reduced IL-2 signaling being associated with reduced Foxp3 expression and Treg stability [56]. The observed differentiation kinetics in vitro potentially mirror both their developmental trajectory and their spatial positioning within the GC in vivo, as the in vivo Tfr cell pool consists of both CD25⁺ and CD25⁻ populations [29]. While CD25^+^ Tfr cells reside primarily in the follicle and the T-B border [14], decreased IL-2 concentrations within the GC may facilitate CD25 downregulation and promote Bcl6 upregulation, thereby stabilizing the mature CXCR5^hi^PD-1^hi^ GC Tfr phenotype [29, 31]. Such fine-tuned regulation of paracrine signals via cytokine gradients within tissues is supported by recent studies using computer simulations and high--resolution imaging [57, 58]. Consistent with this, high IL-2 concentrations at the peak of influenza infection induce Blimp-1 expression in Tregs and prevent Tfr cell development, whereas decreased IL-2 levels during infection resolution are associated with CD25 downregulation and Bcl6 upregulation [46].

We further showed that the transcription factor c-Maf is essential for the acquisition of the follicular phenotype of Tfr cells, which is consistent with previous reports on Tfh and Tfr cells [34, 41, 42, 47]. In the absence of c-Maf, Tfh (TGF-β) cell cultures display increased CD25 expression and reduced CXCR5 expression [34]. Notably, c-Maf also acts as a negative regulator of IL-2 [59], which in turn has been identified as a negative regulator of Tfh cell differentiation [44]. Surprisingly, although it has been previously reported that Tfh cells are resistant to converting into a FoxP3^+^ phenotype [8], we showed that ex vivo-sorted Tfh cells and in vitro-generated Tfh cells could upregulate FoxP3 in the presence of TGF-β and the absence of IL-6/IL-21. This finding overlaps with a recent study by Jacobsen et al., which revealed that FoxP3 expression is upregulated in GC-Tfh cells during the late stage of a GC response but also in Tfh cells cultured in vitro [33]. Importantly, in our experiments, compared with Tfh (TGF-β) cells, CXCR5⁺ Tfr cells also emerged from FoxP3⁺ precursors but with delayed kinetics of CXCR5 upregulation, indicating that follicular programming can proceed despite IL-2–mediated constraints as long as TGF-β is present. One possible explanation for the aforementioned observations is the capacity of Tregs to function as IL-2 sinks [60, 61]. Early during infection and T-cell priming, elevated IL-2 concentrations may not only inhibit the entry of Tregs into the GC but also localize them in close proximity to sites of initial Tfh cell activation, where certain dendritic cell subsets may act as additional IL-2 sinks [62]. As the immune response progresses and IL-2 levels decrease, Tregs may convert into Tfr cells and migrate together with Tfh cells into the follicular environment and subsequently acquire a GC-like phenotype.

A limitation of our in vitro Tfr differentiation system is the intended but simplified physiological context. In contrast to the GC microenvironment, these in vitro cultures do not recapitulate structural and cellular cues such as follicular dendritic cells, antigen availability, or the complex interactions between Tfh cells, B cells, and APCs. Moreover, they may not reproduce spatial gradients of IL-2, IL-21, and chemokines that could shape Tfr cell differentiation and positioning. As our in vitro system employs strong, directed cytokine and costimulatory cues, it may partially bypass the stringent transcriptional requirements observed in vivo. Therefore, while the cell populations generated under these conditions share key phenotypic and functional characteristics with Tfh and Tfr cells, they likely represent in vitro analogs rather than bona fide equivalents, similar to other in vitro-generated T helper cell subsets. Taking these caveats into account, however, in vitro modeling of Tfr cell biology provides novel opportunities for in-depth characterization of this important immune cell type.

## Materials and methods

### Experimental animals

IFNγ-IL-17A-FoxP3 triple-reporter mice were generated by intercrossing GREAT (*Ifng*^*tm3.1Lky*^) [63], Smart-17A (*Il17a*^*tm1.1Lky*^) [64], and *Foxp3*^*hCD2*^ (*Foxp3*^*tm1(CD2/CD52)Shori*^) [65] alleles. *Cd4*-*CreERT2* knock-in (*C57BL/6-CD4*^*tm1(CreERt2)ThBu*^) mice [66] were crossed with *Tgfbr2*^*fl/fl*^ (*Tgfbr2*^*tm1Karl*^) mice [67]. *Maf*^*fl/fl*^ mice [68] were crossed with *Cd4*-*Cre* mice [69]. *Foxp3*^*YFP-Cre*^ (*Foxp3*^*tm4(YFP/icre)Ayr*^) mice [70] were intercrossed with *Rosa26*^*fl-Dtr2AtdTomato*^ mice that were newly generated as previously described [71]. Briefly, the *Dtr2AtdTomato* construct was introduced into the *Rosa26* locus by gene targeting of Bruce4 ES cells, in which expression from the CAG promoter was prevented by loxP and rox-flanked stop cassettes. Upon germline transmission, such mice were intercrossed with Dre deleter mice to generate the *Rosa26*^*fl-Dtr2AtdTomato*^ allele, which served here as a fate-mapping reporter for FoxP3-Cre-activatable tdTomato expression. *Bcl6*^*fl/fl*^ (*Bcl6*^*tm1.1Dent*^, stock number 023727) and *Rosa26*^*fl-Stop-fl-YFP*^ (*Gt(ROSA)26Sor*^*tm1(EYFP)Cos*^, stock number 006148) mice were purchased from The Jackson Laboratory. Wild-type C57BL/6 mice were purchased from Charles River Europe. All animal experiments were performed in accordance with European regulations and federal law of Germany and approved by the Landesamt für Natur-, Umwelt und Verbraucherschutz NRW.

### Cell culture

Peripheral lymph nodes and/or the spleen were isolated and separately minced between the frozen ends of two glass microscope slides. To remove erythrocytes from the lymphoid tissue suspensions, red blood cell lysis was performed, and the resulting cell suspensions were passed through a 70-µm cell strainer (VWR, #732–2758). Naïve CD4^+^ T cells were isolated through negative selection using either the EasySep Mouse Naïve CD4^+^ T-Cell Isolation Kit (STEMCELL Technologies, catalog no. 19765) or the MojoSort Mouse CD4 Naïve T-Cell Isolation Kit (BioLegend, catalog no. 480040), following the protocols provided by the manufacturers; however, half of the recommended antibody and bead volumes were used. After isolation, the viability of the cells was assessed using acridine orange/propidium iodide staining with a LUNA-FX7™ Automated Cell Counter (Logos Biosystems). A total of 4×10^4^ naïve CD4^+^ T cells were cultured in 200 µl of complete medium (RPMI: 10% fetal calf serum (FCS), 10 mM HEPES, 100 U/mL penicillin/streptomycin, 1 mM sodium pyruvate, 1x nonessential amino acid solution, 50 µM β-mercaptoethanol) on plate-bound anti-CD3 (2 µg/ml, clone 145-2C11, Biolegend) and anti-CD28 (2 µg/ml, clone 37.51, Biolegend) in 96-well flat-bottom tissue culture suspension plates (Sarstedt, #83.3924.500) for 3.5 days at 37 °C and 5% CO_2_. The combinations of cytokines and blocking antibodies used in cell culture are listed in Table S1. Where indicated, IL-2 or anti-IL-2 (clone JES6-5H4) was added to the cell cultures. For in vitro generation of tamoxifen-induced conditional knockout alleles, 5×10^5^ purified naïve CD4^+^ T cells isolated from *Cd4-CreERT2*^*+*^ mice were incubated overnight in 1 ml of cRPMI supplemented with 1 µM 4-hydroxytamoxifen (Sigma‒Aldrich) in 12-well flat-bottom tissue culture suspension plates (Sarstedt, #83.3921.500). On the next day, the cells were harvested, washed twice with medium, and plated on anti-CD3/anti-CD28-coated plates as described above.

### Flow cytometry

Flow cytometry was performed as previously described [34, 72, 73]. In brief, the cells were harvested, washed with 1× phosphate-buffered saline (PBS) and transferred to 96-well V-bottom plates. To exclude dead cells, the cells were stained for 15 min on ice with Fixable Viability Dye eFluor™ 780 (eBioscience). To reduce nonspecific binding, the cells were preincubated with a CD16/32 Fc block (clone 93; BioLegend) and normal rat serum (NRS; Stemcell Technologies). For surface staining, the cells were incubated for 30 min on ice with the antibodies listed in Table S2. CXCR5 expression was assessed using a biotinylated anti-CXCR5 antibody (clone L138D7; Biolegend), followed by washing and a 20-min incubation with fluorophore-conjugated streptavidin (SA) on ice. For transcription factor staining, the cells were fixed and permeabilized in Fixation/Permeabilization Buffer for 15 min at room temperature with the eBioscience Foxp3/Transcription Factor Staining Buffer Set (Thermo Fisher Scientific), after which they were washed and stained with antibodies in permeabilization buffer for 45 min. For intracellular cytokine staining, cells were stimulated for 4 h with 20 nM phorbol 12-myristat 13-acetat (PMA; Sigma Aldrich) and 1 µM ionomycin (Sigma Aldrich). After two hours of stimulation, monensin (GolgiStop, BD Biosciences) was added for the remaining incubation period according to the manufacturer’s instructions. The cells were then fixed with 4% paraformaldehyde (PFA; Thermo Fisher) in PBS for 8 min at RT, washed with PBS and subsequently permeabilized on ice for 5 min using buffer containing 0.5% saponin, 1% bovine serum albumin (BSA), and 0.1% sodium azide (NaN_3_) in PBS. Intracellular IL-21 was detected by overnight staining with recombinant IL-21-human-Fc chimera (R&D Systems, #596-MR-100), followed by R-Phycoerythrin AffiniPure® F(ab´)_2_ Fragment Goat Anti-Human IgG Fcγ Fragment (Jackson ImmunoResearch Laboratories Inc., #109-116-098) staining for 45 min on ice. Cell culture samples were acquired using the BD™ High Throughput Sampler (HTS) option for the BD LSRFortessa. Cell sorting was performed on a BD FACSAriaFusion or BD FACSAria III. Data were analyzed with FlowJo software (Treestar) and compensated with OneComp eBeads Compensation Beads (Thermo Fisher). While the data depicted in the representative flow cytometry plots were rounded for display, the underlying data were calculated with higher precision and are depicted in the corresponding statistical analyses.

### B-cell–T-cell coculture

B cells were enriched from the spleens of wild-type C57BL/6 mice with an EasySep™ Mouse B-Cell Isolation Kit (Stemcell Technologies, catalog #19854) following the manufacturer’s protocol. To achieve higher purity, two rounds of isolation were performed. To compare in vitro-generated versus ex vivo-sorted Tfr cells, IFNγ-IL-17A-FoxP3 triple-reporter mice were immunized s.c. in both flanks with 50 µg of ovalbumin (OVA) premixed with Complete Freund’s Adjuvants (CFA; #EK-0301; Hooke Laboratories). After seven days, axillary and inguinal lymph nodes were harvested, and single-cell suspensions were enriched for CD4^+^ T cells using a Biolegend MojoSort Mouse CD4 T-Cell Isolation Kit (catalog #480033). Tfh cells were further purified by flow cytometry-based cell sorting as live CD8α^–^CD19^–^CD4^+^FoxP3^–^CXCR5^+^PD-1^+^ cells, while Tfr cells were sorted as live CD8α^–^CD19^–^CD4^+^FoxP3^+^CXCR5^+^ cells. Ex vivo-sorted Tfr cells were tested for their suppressive capacity together with in vitro-generated Tfr cells in a B-cell‒T-cell coculture assay as previously described [23, 34]. To this end, a total of 5×10^4^ B cells were cocultured together with 3×10^4^ ex vivo-sorted or in vitro-generated Tfh cells in the presence of in vitro-generated or ex vivo-sorted Tfr cells (1.5×10^4^ each) or no additional Tfr cells in complete RPMI supplemented with soluble anti-CD3 (2 µg/ml) and anti-IgM (5 µg/ml). For the analysis of T–B-cell coculture-derived ex vivo-sorted Tfh cells, ex vivo Tfh cells were first sorted from unimmunized CD45.2^+^CD45.1^–^ IFNγ-IL-17A-FoxP3 triple-reporter mice as described above, while in vitro Tfr cells were generated from naïve CD4^+^ T cells isolated from wild-type CD45.2^+^CD45.1^+^ mice. T–B-cell co-cultures were then cultured for 3.5 days at 37 °C with 5% CO₂ and analyzed by flow cytometry thereafter. T-B cell co-cultures with in vitro-differentiated CD4^+^ T cells from *Cd4*-*Cre*^*–*^*. Bcl6*^*fl/fl*^ and *Cd4*-*Cre*^*+*^*. Bcl6*^*fl/fl*^ mice were established in a similar manner as described above.

### In vivo analyses

Axillary, inguinal and/or mesenteric lymph nodes were isolated from unimmunized or OVA/CFA-immunized *Cd4-Cre*^*– mice*^*. Maf*^*fl/fl*^ and *Cd4-Cre*^*+*^*. Maf*^*fl/fl*^ mice or *Cd4-Cre*^*–*^*. Bcl6*^*fl/fl*^ and *Cd4-Cre*^*+*^*. Bcl6*^*fl/fl*^ mice. Peyer’s patches were isolated from nonimmunized *Foxp3*^*YFP-Cre mice*^. *Rosa26*^*fl-Dtr2AtdTomato*^ reporter mice, after which single-cell suspensions were prepared. The cells were stained and analyzed by flow cytometry.

### Bulk RNA sequencing and analyses

Treg, Tfr, and Tfh cell subsets were generated in vitro from naïve CD4^+^ T cells obtained from GREAT.Smart-17A.*FoxP3*^*hCD2*^ donor mice. After 3.5 days of cell culture, T cells were harvested, stained, and sorted on a BD FACSAriaFusion cell sorter for FoxP3^+^CXCR5^–^, FoxP3^+^CXCR5^+^, and FoxP3^–^CXCR5^+^ cells among viable CD8^–^CD19^–^CD4^+^ lymphocytes using a 70-micron nozzle. RNA was isolated using TRIzol™ LS Reagent (Invitrogen, #10296028) according to the manufacturer’s instructions. In brief, the cells were immediately spun down after sorting and resuspended in 0.25 ml of PBS, after which 0.75 ml of TRIzol LS Reagent was added to each sample. After homogenization, 0.2 ml of chloroform was added, and the mixture was incubated for 3 min. The samples were subsequently centrifuged for 15 min at 12,000 × g at 4 °C, after which the aqueous phase containing the RNA was transferred to a new tube. Next, 0.5 ml of isopropanol was added to the aqueous phase and incubated for 10 min. The RNA precipitate formed after centrifugation at 12,000 × g was resuspended in 1 ml of 75% ethanol. After another 5-min centrifugation step at 7500 × g, the RNA pellet was air-dried and resuspended in RNAse-free water. RNA was removed with an RNeasy Mini Kit (catalog #74104) according to the manufacturer’s instructions. The sequencing library was prepared according to the manufacturer’s guidelines with the QuantSeq FWD 3´-mRNA-Seq Kit from Lexogen using 50 ng of total RNA as input. Libraries were single-end sequenced with 1 × 100 bp on an S1 flow cell of an Illumina NovaSeq 6000 instrument to a depth of 10 M raw reads on average at the NGS Core Facility of University Hospital Bonn.

Raw sequencing reads in FASTQ format (single-end) were subjected to quality control using FastQC (v0.12.1). Adapter sequences and low-quality bases were removed with Trimmomatic (v0.39). Clean reads were aligned to the *Mus musculus* reference genome (GRCm39, Ensembl release 104) using HISAT2 (v2.2.1) under default parameters. Read counts per gene were generated using featureCounts (v2.0.1) from the Subread package, which is based on Ensembl gene annotations. Raw count matrices were imported into R (v4.4.2) for downstream analysis. To focus on biologically relevant features, only protein-coding genes were retained, as defined by annotations from the Ensembl BioMart database, reducing the dataset from 20,605 annotated genes to 13,311 protein-coding genes. Genes were further filtered on the basis of minimum expression criteria: a gene was retained if it showed more than five raw counts in at least 75% of samples within at least one experimental group. This filtering step reduced the dataset to 5520 genes, which were used for all downstream analyses. Differential expression analysis was performed using the DESeq2 package (v1.42.0). Count data were modeled with a negative binomial distribution, and normalization was carried out using the median-of-ratios method implemented in DESeq2. Pairwise comparisons between experimental groups (Treg, Tfr, and Tfh) were conducted using the Wald test and multiple-testing correction using the Benjamini‒Hochberg method. Genes were considered differentially expressed if they met both thresholds: a false discovery rate (FDR) < 0.1 and an absolute log2-fold change > 0.5. The sequencing data have been deposited in GEO: GSE306188.

### Statistical analysis

Statistical analyses were performed using Prism 10 (GraphPad), with the specific tests described in the legend of each corresponding figure.

## Supplementary information

Supplementray Material

Supplementary Data 1
