# Supplementary material for: TGF-β and IL-2 differentially shape T follicular regulatory cell differentiation and stability in vitro
Source: Cell Mol Immunol. 2026 Jun 25;23(8):1022–37. doi: 10.1038/s41423-026-01440-9 (PMC13424134; doi:10.1038/s41423-026-01440-9)
Supplement: Supplementary file 1 — Supplementray Material [file 41423_2026_1440_MOESM1_ESM.pdf]

Supplementary Materials for

TGF- β and IL-2 differentially shape T follicular regulatory cell differentiation and stability *in vitro*

Luisa Bach, Yinshui Chang, Olin Arteaga Transito, Mohammadamin Ghasemi, Lisa Maria Steinheuer, Teresa Steffen, Elena De Domenico, Thomas Ulas, F. Thomas Wunderlich, Marc D. Beyer, Kevin Thurley, Dirk Baumjohann*

*Corresponding author: dirk.baumjohann@uni-bonn.de

This PDF includes:

Figures S1 to S8
Tables S1 and S2

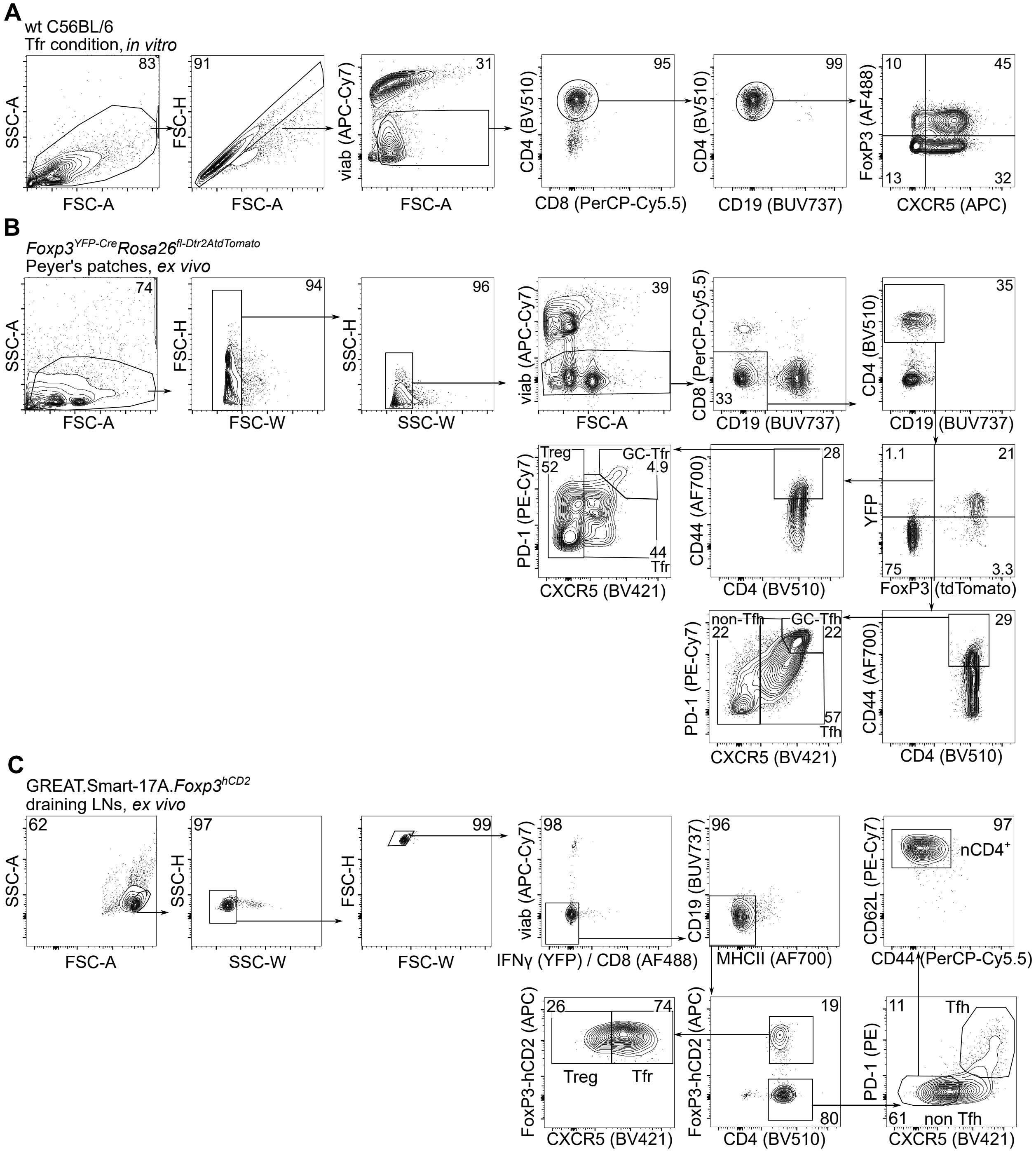

Supplementary Fig. 1. Gating strategies of flow cytometry data.

A Representative gating strategy for the identification of CXCR5 and FoxP3-expressing cells in the *in vitro* T helper cell differentiation cell cultures, in this case a Tfr cell culture.

B Representative gating strategy for the identification of *in vivo* Treg, Tfr, and Tfh cells in the Peyer's patches of *Foxp3*$^{YFP\text{-}Cre}$*.Rosa26*$^{fl\text{-}Dtr2AtdTomato}$ reporter mice.

C Representative gating strategy for the identification and isolation of *ex vivo*-sorted naive CD4^{+} T (nCD4^{+}), Tfh, Treg, and Tfr cells from draining lymph nodes one week after subcutaneous OVA/CFA immunization used for *in vitro* T-B cell co-culture assays and *ex vivo* T cell cultures.

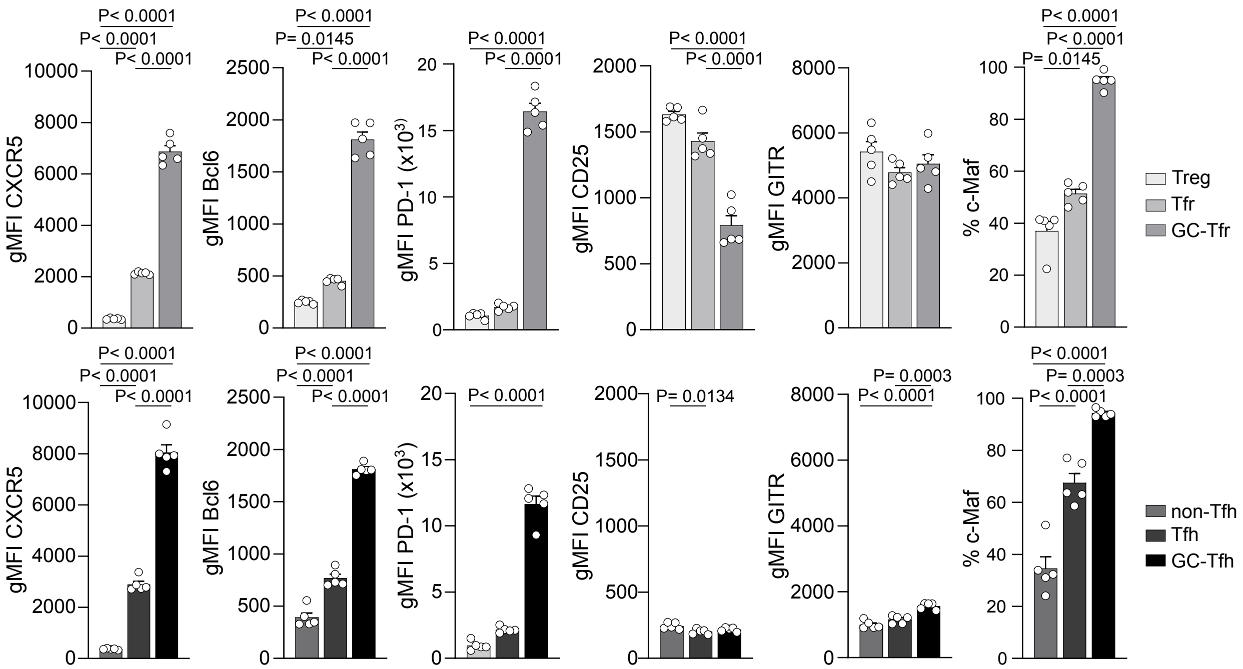

Supplementary Fig. 2. Phenotypic characterization of *in vivo* Treg, Tfr, and Tfh cells. Quantification of the histograms shown in Fig. 1G for CXCR5, Bcl6, PD-1, CD25, GITR, and c-Maf expression in Treg, Tfr, and GC-Tfr (top row) as well as non-Tfh, Tfh, and GC-Tfh cells (bottom row) analyzed from Peyer's patches of *Foxp3*$^{YFP\text{-}Cre}$.*Rosa26*$^{fl\text{-}Dtr2AtdTomato}$ reporter mice. Data are representative of two independent experiments displaying mean ± SEM with n = 5 biological replicates per condition. Ordinary one-way ANOVA with Tukey's multiple comparisons test. Only significant p values < 0.05 are shown.

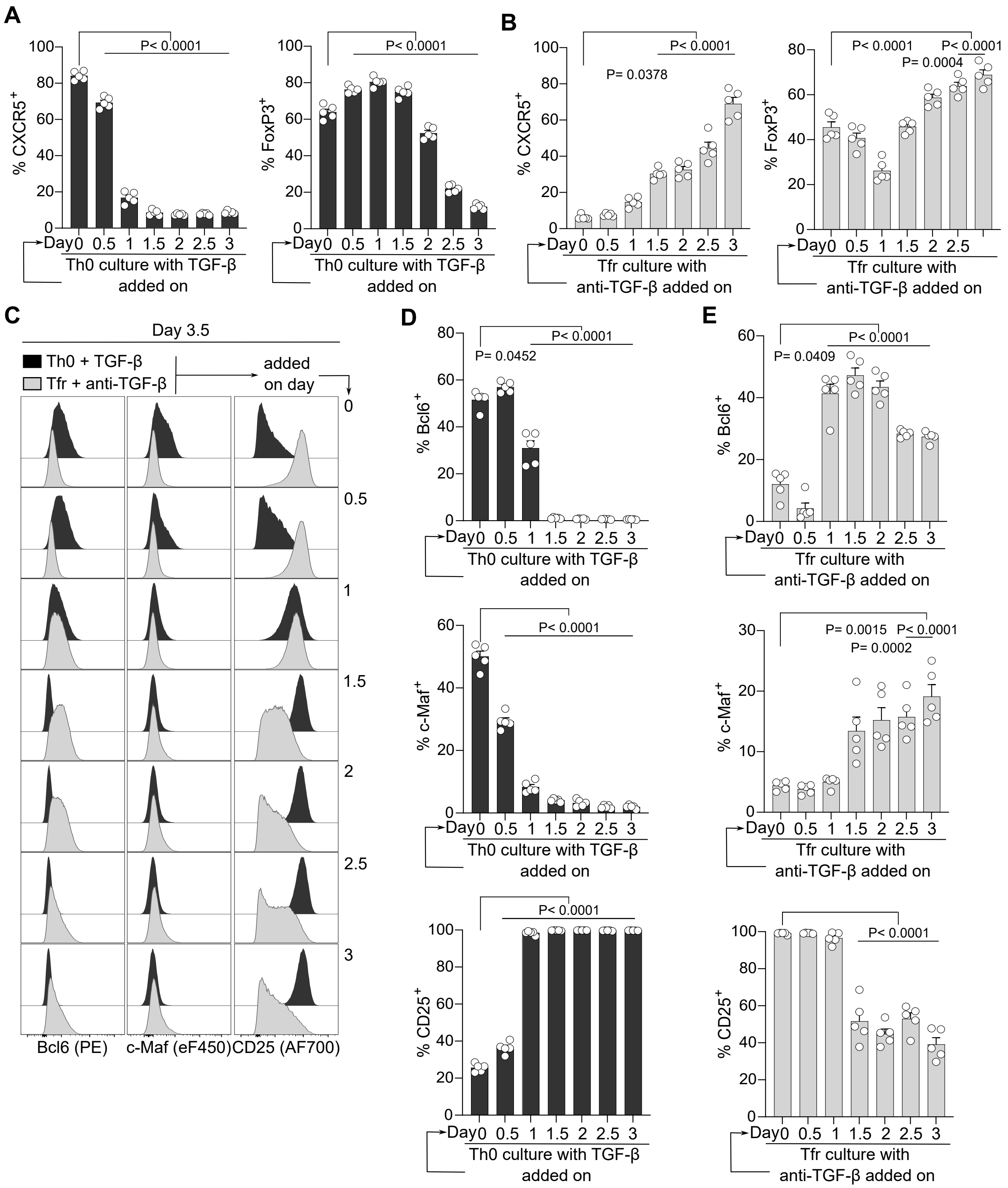

Supplementary Fig. 3. TGF-β is essential at early stages of Tfr cell differentiation

A Quantification of the frequencies of total $CXCR5^+$ and $FoxP3^+$ $CD4^+$ T cells after 3.5 days of *in vitro* Th0 culture with 5 ng/ml TGF-β added at indicated time points, for the experiment displayed in Fig. 4.

B Quantification of the frequencies of total $CXCR5^+$ and $FoxP3^+$ cells after 3.5 days of *in vitro* Tfr culture with 10 μg/ml anti-TGF-β added at indicated time points, for the experiment displayed in Fig. 4.

C Representative histograms of Bcl6, CD25, and c-Maf expression in Th0 cultures with 5 ng/ml TGF-β or Tfr cultures with 10 μg/ml anti-TGF-β added at indicated time points, for the experiment displayed in Fig. 4.
D Quantification of frequencies of Bcl6^{+}, c-Maf^{+}, and CD25^{+} cells after 3.5 days of *in vitro* Th0 culture with 5 ng/ml TGF-β added at indicated time points, for the experiment displayed in Fig. 4.
E Quantification of frequencies of Bcl6^{+}, c-Maf^{+}, and CD25^{+} cells after 3.5 days of *in vitro* Tfr culture with 10 μg/ml anti-TGF-β added at indicated time points, for the experiment displayed in Fig. 4.
Data are representative of three independent experiments displaying mean ± SEM with n = 5 biological replicates per condition. Ordinary one-way ANOVA with Dunnett's multiple comparisons test against day 0. Only significant p values < 0.05 are shown.

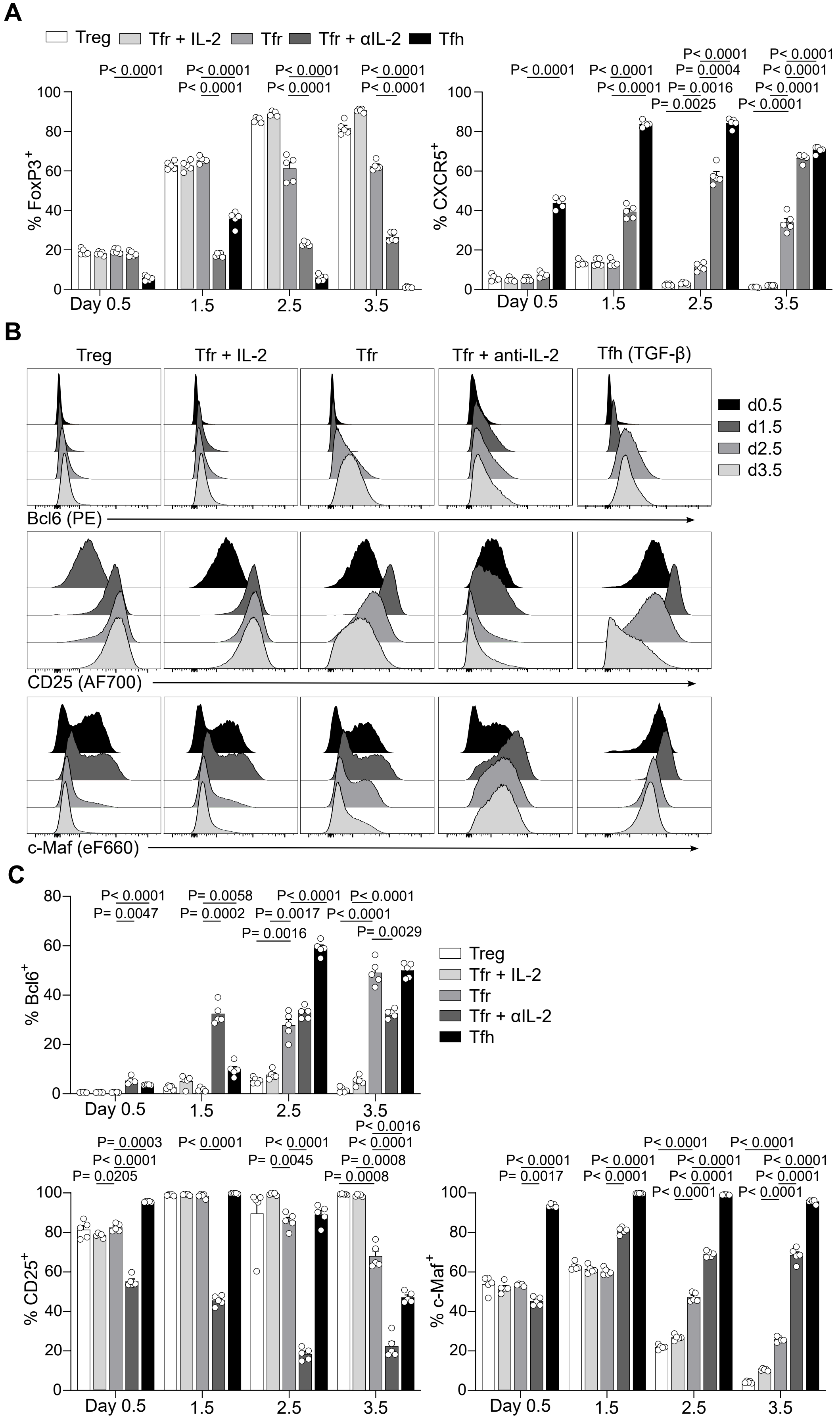
A
Treg
Tfr + IL-2
Tfr
Tfr + αIL-2
Tfh
% FoxP3+
% CXCR5+
Day 0.5
1.5
2.5
3.5
B
Treg
Tfr + IL-2
Tfr
Tfr + anti-IL-2
Tfh (TGF-β)
d0.5
d1.5
d2.5
d3.5
Bcl6 (PE)
CD25 (AF700)
c-Maf (eF660)
C
% Bcl6+
% CD25+
% c-Maf+

Supplementary Fig. 4. Sequential expression of Treg and Tfh-associated markers define the developmental trajectory of Tfr cells

A Quantification of the frequencies of $CXCR5^+$ and $FoxP3^+$ cells after 0.5, 1.5, 2.5, and 3.5 days of *in vitro* Treg, Tfr or Tfh (TGF-β) culture. Where indicated, 40 ng/ml IL-2 or 10 μg/ml anti-IL-2 were added at the start of the Tfr culture. Supplementary data for the experiment displayed in Fig. 5.

B Representative histograms of Bcl6, CD25, and c-Maf expression after 0.5, 1.5, 2.5, and 3.5 days of *in vitro* Treg, Tfr or Tfh (TGF-β) culture. Supplementary data for the experiment displayed in Fig. 5.

C Quantification of frequencies of $Bcl6^+$, c-Maf^+, and $CD25^+$ cells after 0.5, 1.5, 2.5, and 3.5 days of *in vitro* Treg, Tfr or Tfh (TGF-β) culture. Supplementary data for the experiment displayed in Fig. 5.

Data are representative of four independent experiments displaying mean ± SEM with n = 5 biological replicates per condition. Two-way ANOVA with Dunnett's multiple comparisons test against Tfr. Only significant p values < 0.05 are shown.

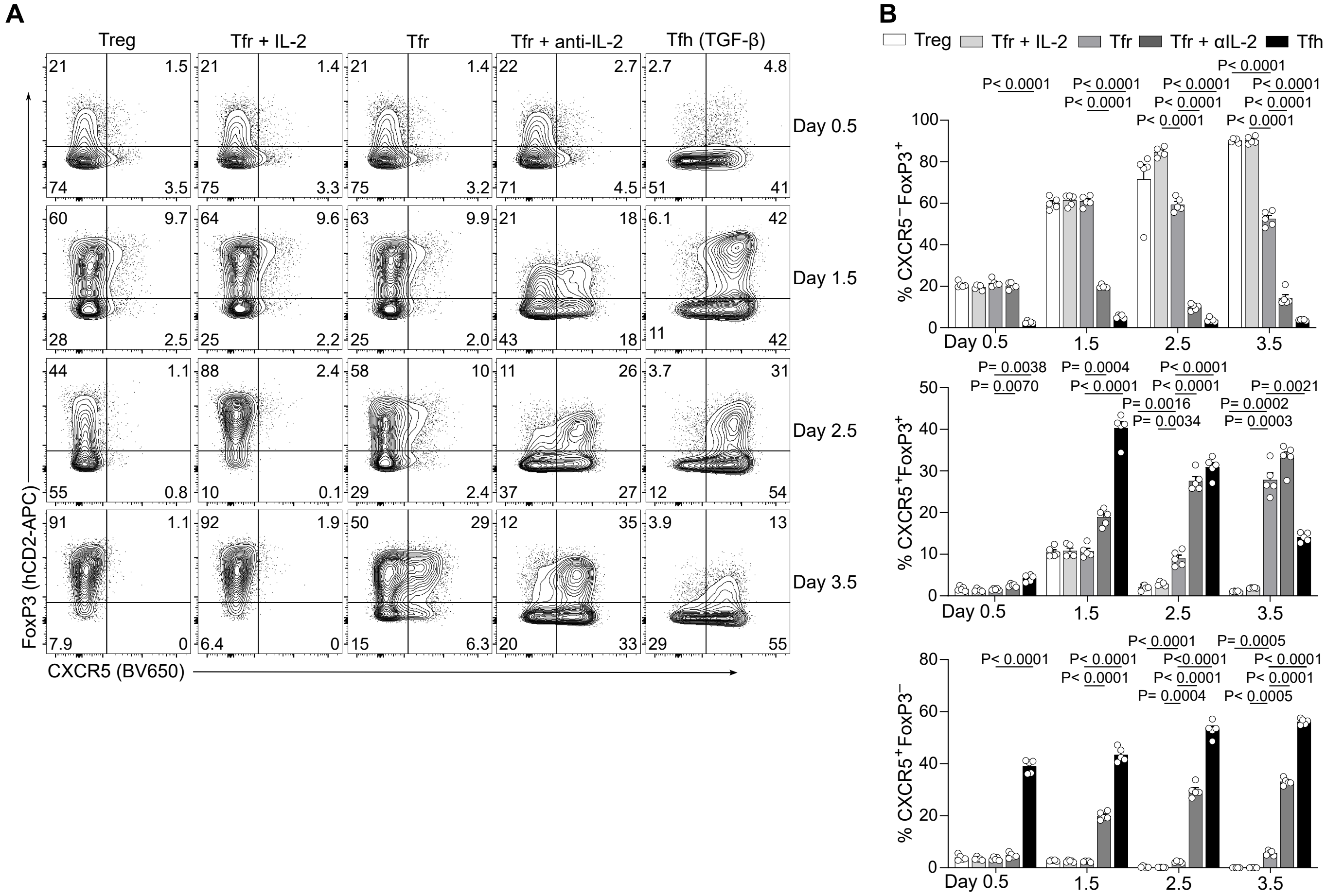

Supplementary Fig. 5. Stepwise differentiation of Tfr cells from FoxP3⁺ precursors.

A Representative flow cytometry contour plots gated on live CD4⁺ T cells stained for CXCR5 and FoxP3 (detected via the hCD2 surface reporter) after 0.5, 1.5, 2.5, and 3.5 days of *in vitro* Treg, Tfr, or Tfh (TGF-β) culture. Where indicated, 40 ng/ml IL-2 or 10 μg/ml anti-IL-2 were added to the Tfr culture. Supplementary data for the same experiment displayed in Fig. 5

B Quantification of the frequencies of CXCR5⁻FoxP3⁺, CXCR5⁺FoxP3⁺, and CXCR5⁺FoxP3⁻ cells in Treg, Tfr, or Tfh (TGF-β) cultures. Supplementary data for the same experiment displayed in Fig. 5.

Data are representative of four independent experiments, respectively with 5 biological replicates per condition. **B** shows mean ± SEM. Two-way ANOVA with Dunnett's multiple comparisons test against the Tfr condition. Only significant p values < 0.05 are shown.

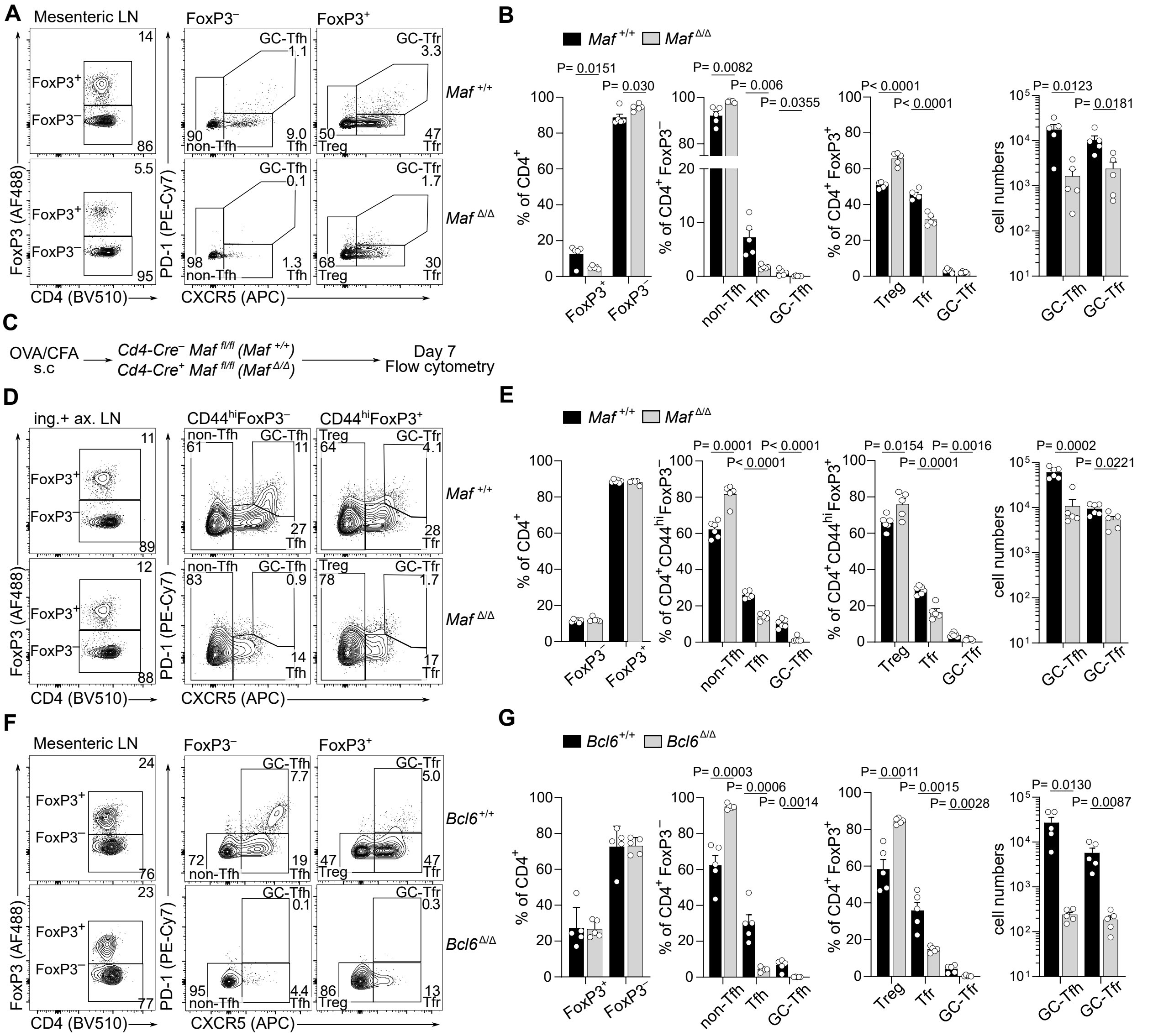

Supplementary Fig. 6. Common transcriptional regulators control Tfh and Tfr cells *in vivo*.
A Representative flow cytometry contour plots from mesenteric LNs of uninfected *Cd4-Cre*$^{–}$ *Maf*$^{fl/fl}$ (*Maf*$^{+/+}$) and *Cd4-Cre*$^{+}$ *Maf*$^{fl/fl}$ (*Maf*$^{Δ/Δ}$) mice showing live CD8$^{–}$CD19$^{–}$CD4^{+}FoxP3^{+} or CD8$^{–}$CD19$^{–}$CD4^{+}FoxP3$^{–}$ T cells stained for CXCR5 and PD-1.
B Frequencies of FoxP3^{+} and FoxP3$^{–}$ cells and frequencies of FoxP3$^{–}$CXCR5$^{–}$non-Tfh cells, FoxP3$^{–}$CXCR5^{+}PD-1$^{low/int}$ Tfh cells, and FoxP3$^{–}$CXCR5^{+}PD-1^{hi} GC-Tfh cells as well as FoxP3^{+}CXCR5$^{–}$ Tregs, FoxP3^{+}CXCR5^{+}PD-1$^{low/int}$ Tfr cells, and FoxP3^{+}CXCR5^{+}PD-1^{hi} GC-Tfr cells are quantified in the bar graph next to the quantification of total GC-Tfh and GC-Tfr cell counts.
C Experimental outline: *Cd4-Cre*$^{–}$ *Maf*$^{fl/fl}$ (*Maf*$^{+/+}$) and *Cd4-Cre*$^{+}$ *Maf*$^{fl/fl}$ (*Maf*$^{Δ/Δ}$) mice were immunized subcutaneously with OVA/CFA. After 7 days, draining inguinal and axillary LNs were analyzed by flow cytometry.
D Representative flow cytometry contour plots of live *Maf*$^{+/+}$ and *Maf*$^{Δ/Δ}$ CD8$^{–}$CD19$^{–}$CD4^{+}CD44^{hi}FoxP3^{+} or CD8$^{–}$CD19$^{–}$CD4^{+}CD44^{hi}FoxP3$^{–}$ T cells from draining inguinal and axillary LNs stained for CXCR5 and PD-1.

E Frequencies of $FoxP3^{+}$ and $FoxP3^{–}$ cells and frequencies of $FoxP3^{–}CD44^{hi}CXCR5^{–}$ non-Tfh cells, $FoxP3^{–}CD44^{hi}CXCR5^{+}PD\text{-}1^{low/int}$ Tfh cells, and $FoxP3^{–}CD44^{hi}CXCR5^{+}PD\text{-}1^{hi}$ GC-Tfh cells as well as $FoxP3^{+}CD44^{hi}CXCR5^{–}$ Tregs, $FoxP3^{+}CD44^{hi}CXCR5^{+}PD\text{-}1^{low/int}$ Tfr cells, and $FoxP3^{+}CD44^{hi}CXCR5^{+}PD\text{-}1^{hi}$ GC-Tfr cells are quantified in the bar graph next to the quantification of total GC-Tfh and GC-Tfr cell counts.
F Representative flow cytometry contour plots of live $Bcl6^{+/+}$ and $Bcl6^{\Delta/\Delta}$ $CD8^{–}CD19^{–}CD4^{+}FoxP3^{+}$ or $CD8^{–}CD19^{–}CD4^{+}FoxP3^{–}$ T cells from uninfected mesenteric LN stained for CXCR5 and PD-1.
G Frequencies of $FoxP3^{+}$ and $FoxP3^{–}$ cells and frequencies of $FoxP3^{–}CXCR5^{–}$ non-Tfh cells, $FoxP3^{–}CXCR5^{+}PD\text{-}1^{low/int}$ Tfh cells, and $FoxP3^{–}CXCR5^{+}PD\text{-}1^{hi}$ GC-Tfh cells as well as $FoxP3^{+}CXCR5^{–}$ Tregs, $FoxP3^{+}CXCR5^{+}PD\text{-}1^{low/int}$ Tfr cells, and $FoxP3^{+}CXCR5^{+}PD\text{-}1^{hi}$ GC-Tfr cells are quantified in the bar graph next to the quantification of total GC-Tfh and GC-Tfr cell counts.
Data in **A+B**, **C-E**, and **F+G** are representative of three, two, and two independent experiments displaying mean ± SEM with n = 5 biological replicates per condition. Multiple unpaired t-tests where only significant p values < 0.05 are shown.

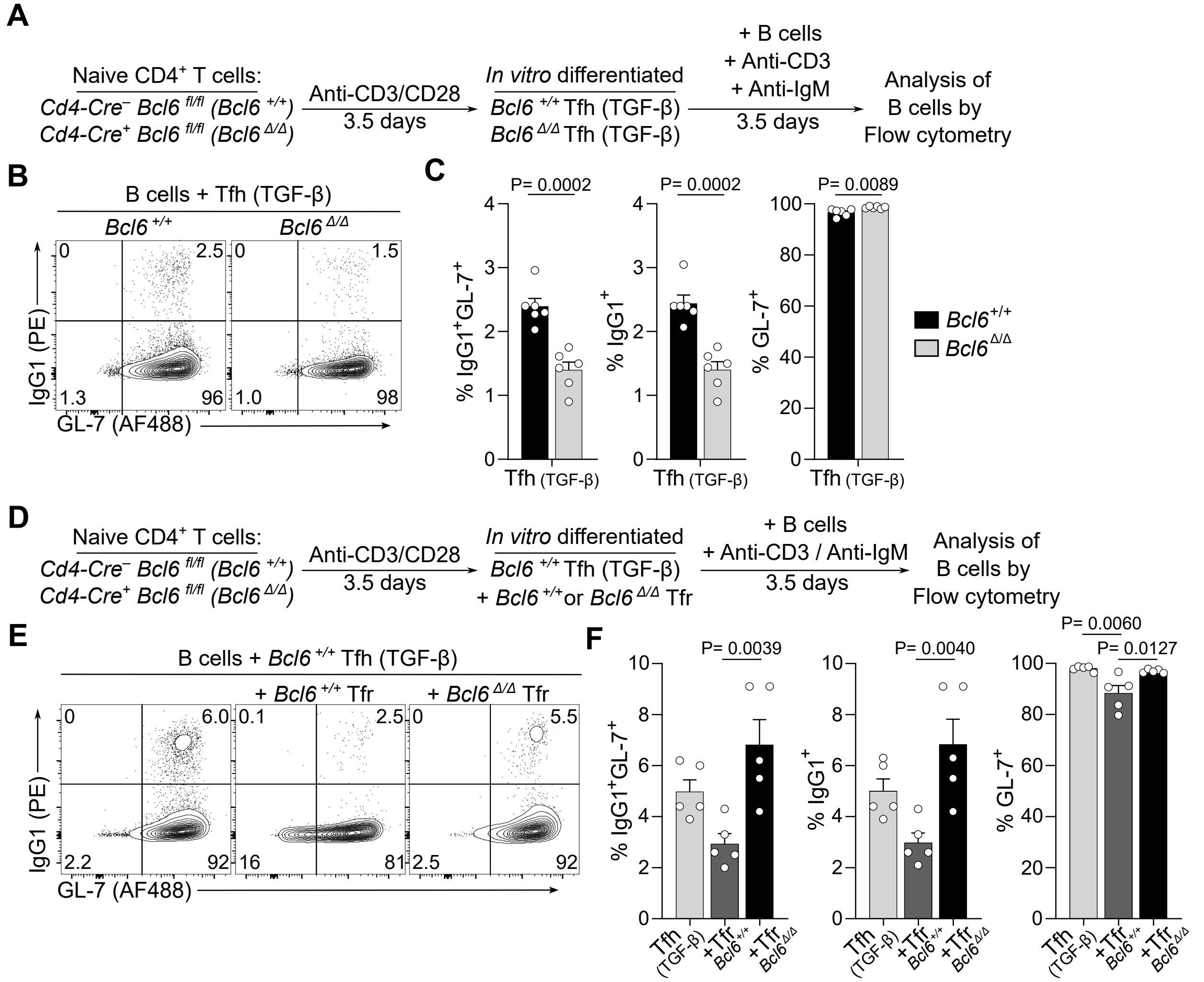

Supplementary Fig. 7. Bcl6 is required for optimal Tfh cell helper function and Tfr cell-mediated suppression.

A Experimental outline: Naïve CD4^{+} T cells from *Cd4-Cre^{-} Bcl6$^{fl/fl}$* (*Bcl6 $^{+/+}$*) and *Cd4-Cre^{+} Bcl6 $^{fl/fl}$* (*Bcl6 $^{\Delta/\Delta}$*) mice were differentiated *in vitro* towards Tfh (TGF-β) cells for 3.5 days and where then tested in co-cultures with purified wildtype C57BL/6 B cells. After 3.5 days, the cultured B cells were analyzed by flow cytometry.

B Flow cytometry contour plots showing the frequency of GL-7^{+}IgG1^{+} B cells after 3.5 days of co-culture with *in vitro*-generated *Bcl6$^{+/+}$* or *Bcl6*-deficient (*Bcl6 $^{\Delta/\Delta}$*) Tfh (TGF-β) cells.

C Quantification of data in **B**.

D Experimental outline: Naïve CD4^{+} T cells from *Cd4-Cre^{-} Bcl6$^{fl/fl}$* (*Bcl6 $^{+/+}$*) and *Cd4-Cre^{+} Bcl6 $^{fl/fl}$* (*Bcl6 $^{\Delta/\Delta}$*) were differentiated *in vitro* towards *Bcl6 $^{+/+}$* Tfh (TGF-β) as well as *Bcl6 $^{+/+}$* or *Bcl6 $^{\Delta/\Delta}$* Tfr cells for 3.5 days and where then tested in co-cultures with purified wild-type C57BL/6 B cells. After 3.5 days, the cultured B cells were analyzed by flow cytometry.

E Flow cytometry contour plots showing the frequency of GL-7^{+}IgG1^{+} B cells after 3.5 days of co-culture with *in vitro*-generated Bcl6$^{+/+}$ Tfh (TGF-β) cells alone or together with *in vitro*–differentiated *Bcl6$^{+/+}$* or *Bcl6 $^{\Delta/\Delta}$* Tfr cell populations. Gated on live CD19^{+}CD4^{-} cells.

F Quantification of data in **E**.

Data are representative of three independent experiments displaying mean ± SEM with n = 3-5 biological replicates per condition. Unpaired t-tests **C** and Ordinary one-way ANOVA with Tukey's multiple comparisons test **F** where only significant p values < 0.05 are shown.

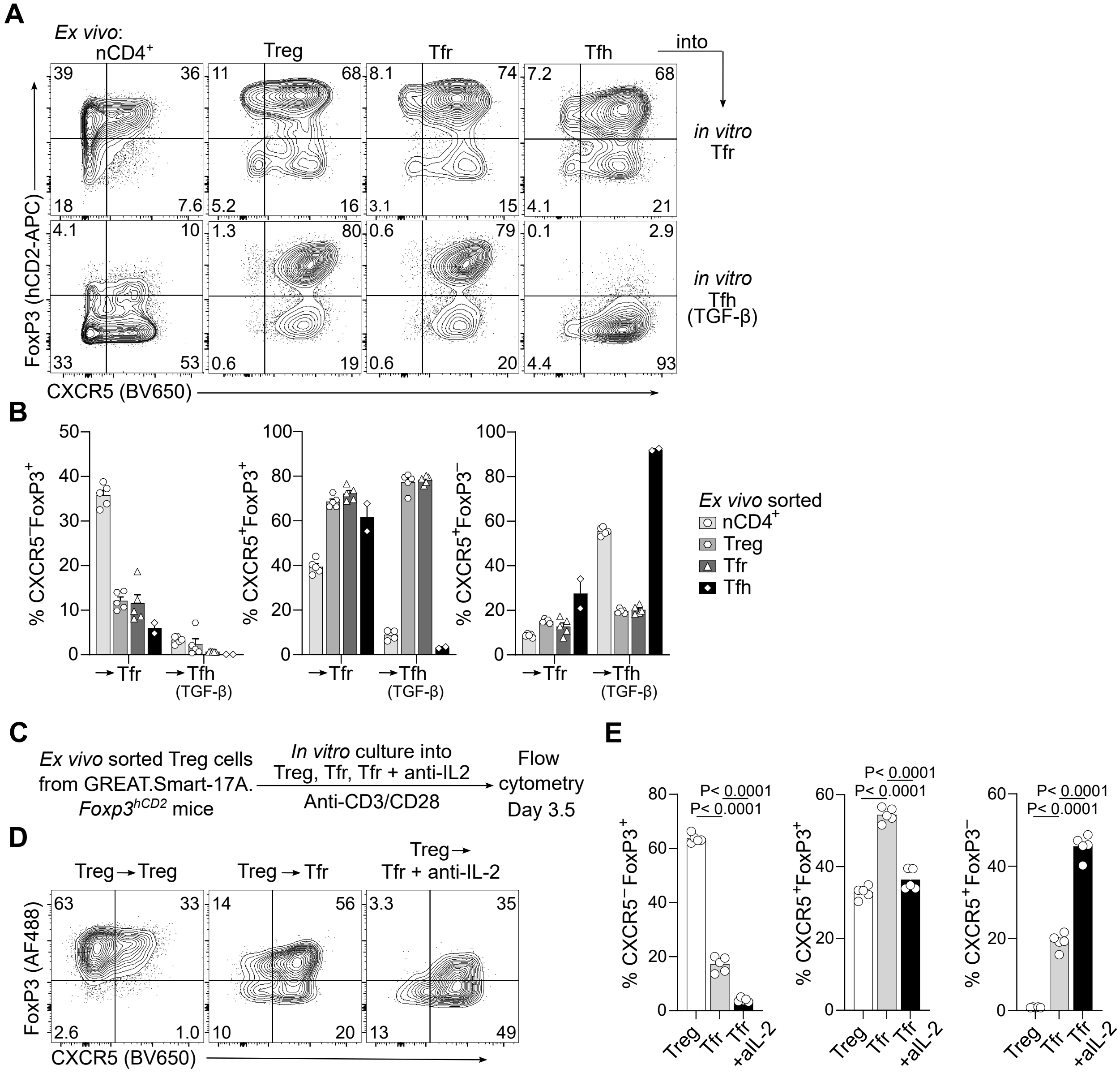

Supplementary Fig. 8. Stability and plasticity of Treg, Tfr, and Tfh cells.

A GREAT.Smart-17A.*FoxP3*^{hCD2} triple-reporter mice were immunized subcutaneously with OVA/CFA and indicated cell populations were sorted and further cultivated *in vitro* in anti-CD3/CD28-coated cell culture plates for 3.5 days under Tfr or Tfh (TGF-β) polarizing conditions as described in Fig. 8A. Representative flow cytometry contour plots gated on live $CD4^+$ T cells stained for CXCR5 and FoxP3 (detected via the hCD2 surface reporter) as opposed to direct intracellular FoxP3 staining shown in Fig. 8B for the same samples.

B Quantification of the data in **A**.

C Experimental outline: $CD4^+CXCR5^-FoxP3^+$ regulatory T cells from GREAT.Smart-17A.*FoxP3*^{hCD2} triple-reporter mice were sorted and further cultivated *in vitro* in anti-CD3/CD28-coated cell culture plates for 3.5 days under Treg or Tfr polarizing conditions. Where indicated, 10 µg/ml anti-IL-2 were added to the Tfr culture.

D Representative flow cytometry contour plots gated on live $CD4^+$ T cells.

E Quantification of the data in **D**.

Data in **A+B** and **D+E** are representative of three and two independent experiments, respectively, displaying mean ± SEM with n = 2-5 biological replicates per condition. Ordinary one-way ANOVA with Dunnett's multiple comparisons test against the Tfr condition **F** where only significant p values < 0.05 are shown.

Table S1. List of reagents for T helper cell cultures

Subset	Cytokine or blocking antibody	Concentration	Source	Clone	Cat #
Th0	anti-IL-4	10 µg/ml	Biolegend	11B11	504122
	anti-IFNγ	10 µg/ml	Biolegend	XMG1.2	505834
Treg	anti-IL-4	10 µg/ml	Biolegend	11B11	504122
	anti-IFNγ	10 µg/ml	Biolegend	XMG1.2	505834
	IL-2	80 ng/ml	Biolegend	NA	575406
	hTGFβ1	2 ng/ml	Peprotech	NA	100-21-10UG
Tfr	anti-IL-4	10 µg/ml	Biolegend	11B11	504122
	anti-IFNγ	10 µg/ml	Biolegend	XMG1.2	505834
	hTGFβ1	5 ng/ml	Peprotech	NA	100-21-10UG
Tfh (TGF-β)	anti-IL-4	10 µg/ml	Biolegend	11B11	504122
	anti-IFNγ	10 µg/ml	Biolegend	XMG1.2	505834
	IL-6	50 ng/ml	Biolegend	NA	575706
	IL-21	25 ng/ml	Biolegend	NA	574504
	hTGFβ1	5 ng/ml	Peprotech	NA	100-21-10UG
Tfh (αTGF-β)	anti-IL-4	10 µg/ml	Biolegend	11B11	504122
	anti-IFNγ	10 µg/ml	Biolegend	XMG1.2	505834
	anti-TGFβ	10 µg/ml	BioXCell	1D11.16.8	BE0057
	IL-6	50 ng/ml	Biolegend	NA	575706
	IL-21	25 ng/ml	Biolegend	NA	574504

Table S2. List of antibodies used for flow cytometry

Marker	Fluorochrome	Source	Clone	Cat #	RRID
CD4	BV510	Biolegend	RM4-5	100559	AB_2561388
CD4	PE	Biolegend	RM4-5	100512	AB_312714
CD8a	PerCP-Cy5.5	Biolegend	53-6.7	100734	AB_2075239
CD8	PE-Cy7	Biolegend	52-6.7	100722	AB_312760

CD16/CD32	-	Biolegend	93	101302	AB_312800
CD19	BUV737	BD Biosciences	3D1	612781	AB_2870111
CD25	AF700	Biolegend	PC61	102024	AB_493709
CD44	PerCP-Cy5.5	Biolegend	IM7	103032	AB_2076204
CD44	AF700	Biolegend	IM7	103026	AB_493713
CD45.1	PerCP-Cy5.5	Biolegend	A20	110728	AB_893346
CD45.2	BV421	Biolegend	104	109832	AB_2565511
CD62L	PE-Cy7	Tonbo Bioscience	MEL-14	60-0621	AB_2621855
CD80	PerCP-Cy5.5	Biolegend	16-10A1	104722	AB_2291392
CD138	APC	BD Biosciences	281-2	558626	AB_1645216
CXCR5	biotinylated	Biolegend	L138D7	145510	AB_2562125
GL7	AF488	eBioscience	GL-7	53-5902-82	AB_2016717
hCD2	APC	Biolegend	RPA-2.10	300214	AB_10895925
I-A/I-E	AF700	Biolegend	M5/114.15.2	107622	AB_493727
IgG1	PE	Biolegend	RMG1-1	406608	AB_10551439
PD-1	BV711	Biolegend	29F.1A12	135231	AB_2566158
PD-1	PE-Cy7	eBioscience	J43	25-9985-82	AB_10853805
PD-1	PE	eBioscience	J43	12-9985-82	AB_466295
PD-L1	BV421	Biolegend	10F.9G2	124315	AB_10897097
GITR	BV711	BD Biosciences	DTA-1	563390	AB_2738176
Streptavidin	APC	Biolegend	-	405207	-
Streptavidin	BV421	Biolegend	-	405226	-
Streptavidin	BV650	Biolegend	-	405231	-
FoxP3	AF488	eBioscience	FJK-16s	53-5773-82	AB_763537
FoxP3	BV421	eBioscience	FJK-16s	404-5773-82	AB_2925536
Bcl6	PE	BD Biosciences	K112-91	561522	AB_10717126
c-Maf	eFluor450	eBioscience	sym0F1	48-9855-41	AB_2762608
c-Maf	eFluor660	eBioscience	sym0F1	50-9855-82	AB_2574388
c-Maf	PE-Cy7	eBioscience	sym0F1	25-9855-82	AB_2811795
IL-4	PE-Cy7	Biolegend	11B11	504117	AB_10895747
